# Machine learning identifies microbiome and clinical predictors of sustained weight loss following prolonged fasting

**DOI:** 10.1186/s13073-026-01765-0

**Published:** 2026-09-16

**Authors:** Gelsomina Kaufhold, Theda U. P. Bartolomaeus, Kristin Schütte, Till Schütte, Sakshi Kamboj, Ulrike Löber, Gabriele Rahn, Victoria McParland, Lena Braun, Lajos Markó, Matanat Mammadli, Alexander Krannich, Lina S. Bahr, Friederike Gutmann, Friedemann Paul, Quinten R. Ducarmon, Georg Zeller, Robin Mesnage, Nicola Wilck, Alma Zernecke, Peter J. Oefner, Wolfram Gronwald, Dominik N. Müller, Sofia K. Forslund-Startceva, Sylvia Bähring, Hendrik Bartolomaeus, Nadja Siebert

**Affiliations:** 1 Max Delbrück Center for Molecular Medicine in the Helmholtz Association (MDC), Berlin, Germany; 2 Experimental and Clinical Research Center (ECRC), A Cooperation of Charité - Universitätsmedizin Berlin and Max Delbrück Center for Molecular Medicine, Berlin, Germany; 3 Charité-Universitätsmedizin Berlin, Corporate Member of Freie Universität Berlin and Humboldt-Universität zu Berlin, Berlin, Germany; 4 DZHK (German Centre for Cardiovascular Research), Partner Site Berlin, Berlin, Germany; 5 Centre for Ecological and Evolutionary Synthesis (CEES), Department of Biosciences, University of Oslo, Oslo, Norway; 6 Institute of Functional Genomics, University of Regensburg, Regensburg, Germany; 7 Department of Nephrology and Internal Intensive Care Medicine, Charité – Universitätsmedizin Berlin, Berlin, Germany; 8 BioStats GmbH, Nauen, Germany; 9 Molecular Systems Biology Unit, European Molecular Biology Laboratory (EMBL), Heidelberg, Germany; 10 Leiden University Center for Infectious Diseases (LUCID), Leiden University Medical Center (LUMC), Leiden, Netherlands; 11 Science Department, Buchinger Wilhelmi Clinic, Überlingen, Germany; 12 Department of Nutritional Sciences, School of Life Course Sciences, Faculty of Life Sciences and Medicine, King’s College London, London, UK; 13 Institute of Experimental Biomedicine, University Hospital Würzburg, Josef-Schneider-Str. 2, 97080 Würzburg, Germany; 14 Cluster of Excellence ImmunoPreCept, Berlin, Germany

**Keywords:** Fasting, Gut microbiome, Weight loss, Machine learning, Personalized nutrition, Precision medicine, Multi-omics, Prevention, Metabolic health

## Abstract

**Background:**

Prolonged fasting may improve metabolic health, but controlled data in healthy adults with longer follow-up and multi-omics profiling are limited. We investigated the immediate and 12-week follow-up effects of a 5-day fasting intervention on body composition, gut microbiome, and circulating and fecal metabolites, and assessed whether baseline characteristics predict individual weight-loss response.

**Methods:**

In a randomized, waitlist-controlled trial, 38 healthy adults completed a 5-day fasting intervention with 12-week follow-up (LEANER study). Outcomes included body mass index and body composition, gut microbiome composition, and plasma and fecal metabolites. Changes over time and between groups were evaluated using regression-based models and paired non-parametric tests, as appropriate. Additionally, permutation-based multivariate testing was performed on microbiome and metabolome data. Twelve-week body weight response was predicted using data-driven machine learning with cross-validation, followed by external validation in three independent cohorts undergoing prolonged fasting protocols.

**Results:**

Fasting reduced body mass index acutely, predominantly driven by loss of fat mass, and these improvements partially persisted at 12 weeks. Fasting induced marked shifts in gut microbiome composition and in plasma and fecal metabolites. Post-fasting and longer-term changes in microbial diversity were associated with baseline microbiome diversity. A model combining baseline microbiome and clinical variables predicted body mass index response at 12 weeks; prominent predictors included an unclassified *Faecalibacterium* species, *Oscillibacter* sp. 50_27, low-density lipoprotein cholesterol, and systolic blood pressure. The model generalized to three independent cohorts, including individuals with metabolic syndrome, patients with multiple sclerosis exposed to repeated fasting, and healthy volunteers fasting for 6–12 days.

**Conclusions:**

In healthy adults, a 5-day prolonged fasting intervention produces robust short-term metabolic changes with partial persistence and consistent remodeling of the gut microbiome and metabolite profiles. Baseline microbiome and clinical characteristics can help stratify expected longer-term responses, supporting the development of individualized fasting-based interventions.

**Trial registration:**

ClinicalTrials.gov, NCT04452916. Prospectively registered on June 29, 2020.

**Supplementary Information:**

The online version contains supplementary material available at https://doi.org/10.1186/s13073-026-01765-0.

## Background

Fasting interventions have gained renewed attention for their potential to modulate metabolism, reshape the gut microbiome, and promote healthy aging [1]. While fasting protocols vary widely from time-restricted eating to fasting-mimicking diets [2], and prolonged food abstinence lasting up to several weeks (up to 26 days) [3], the underlying principle is consistent: transient caloric deprivation triggers coordinated physiological responses that may confer long-term health benefits [4].

Continuous energy restriction remains the most established dietary strategy for weight loss, whereas intermittent fasting encompasses distinct approaches, including time-restricted eating, alternate-day fasting, and whole-day fasting [4]. Randomized studies indicate that these interventions can reduce body weight and improve selected cardiometabolic risk factors, although intermittent fasting regimens have not consistently demonstrated greater efficacy than continuous energy restriction [5, 6]. By contrast, prolonged fasting over several consecutive days remains less well characterized in randomized controlled studies, particularly in healthy adults with longitudinal follow-up and integrated molecular phenotyping.

Evidence from patient populations, including those with metabolic syndrome [7], autoimmune diseases [8, 9], and cancer [10], suggests that fasting can influence key regulatory pathways such as autophagy, insulin sensitivity, and oxidative stress [1]. In individuals with metabolic syndrome, we previously demonstrated that a 5-day fast atop a DASH (Dietary Approaches to Stop Hypertension) diet induced profound and coordinated changes in gut microbiome composition and immune cell states most of which were reversed at 12 weeks follow-up [7]. Using immune cell population or microbiome profiles, we developed models that predicted sustained antihypertensive effects, supporting the concept of data-driven stratification [5].

Metabolic effects of weight loss interventions such as fasting may be assessed by determination of resting energy expenditure (REE). REE is defined as the energy required to sustain vital physiological functions while at rest and without any external activity or food intake [11]. It accounts for approximately 60–75% of total daily energy expenditure and is the most significant contributor to overall metabolism [11]. The value is influenced by factors including age, sex, genetics, and body composition, with lean muscle mass being a dominant determinant [12]. Changes in REE are central to understanding metabolic adaptations and the effects of interventions, whether nutritional, pharmacological, or involving lifestyle changes. Reliable measurement is typically achieved through indirect calorimetry, considered the gold standard [13, 14]. Changes in REE may reflect body responses to interventions and contribute to understanding individual variability in weight management and energy requirements.

In parallel, gut microbiome profiling has emerged as a powerful tool for personalized medicine [15]. Microbial signatures have been linked to energy metabolism [16], weight regulation [17, 18], and immune modulation [19, 20]. Personalized dietary approaches that account for microbiome composition have demonstrated superior outcomes in metabolic control [8]. Emerging applications extend to autoimmune, neuropsychiatric, and oncologic indications, underscoring the potential of microbiome-informed strategies in precision medicine [15, 21].

Despite these advances, the long-term effects of prolonged fasting in healthy individuals remain poorly characterized. Most existing studies rely on non-randomized designs, lack adequate control groups, or fail to include follow-up assessments. Furthermore, many interventions are performed in multimodal clinical settings involving exercise, psychological support, and dietary coaching [7], making it difficult to isolate the effect of fasting alone.

Here, we present a randomized, waitlist-controlled study investigating the effects of a 5-day prolonged fasting intervention in healthy adults, with follow-up assessments at 12 weeks. We integrated clinical, microbiome, and metabolome profiling to characterize the immediate and 12-week follow-up physiological responses to prolonged fasting. Using machine learning (ML), we show that sustained weight loss can be predicted from baseline microbiome and clinical features. Results were validated in one independent healthy cohort and cohorts with metabolic syndrome and multiple sclerosis. Our findings offer mechanistic insight into fasting responses and support the potential of microbiome-informed strategies for personalized lifestyle interventions.

## Methods

### Study design

**Fig. 1:**
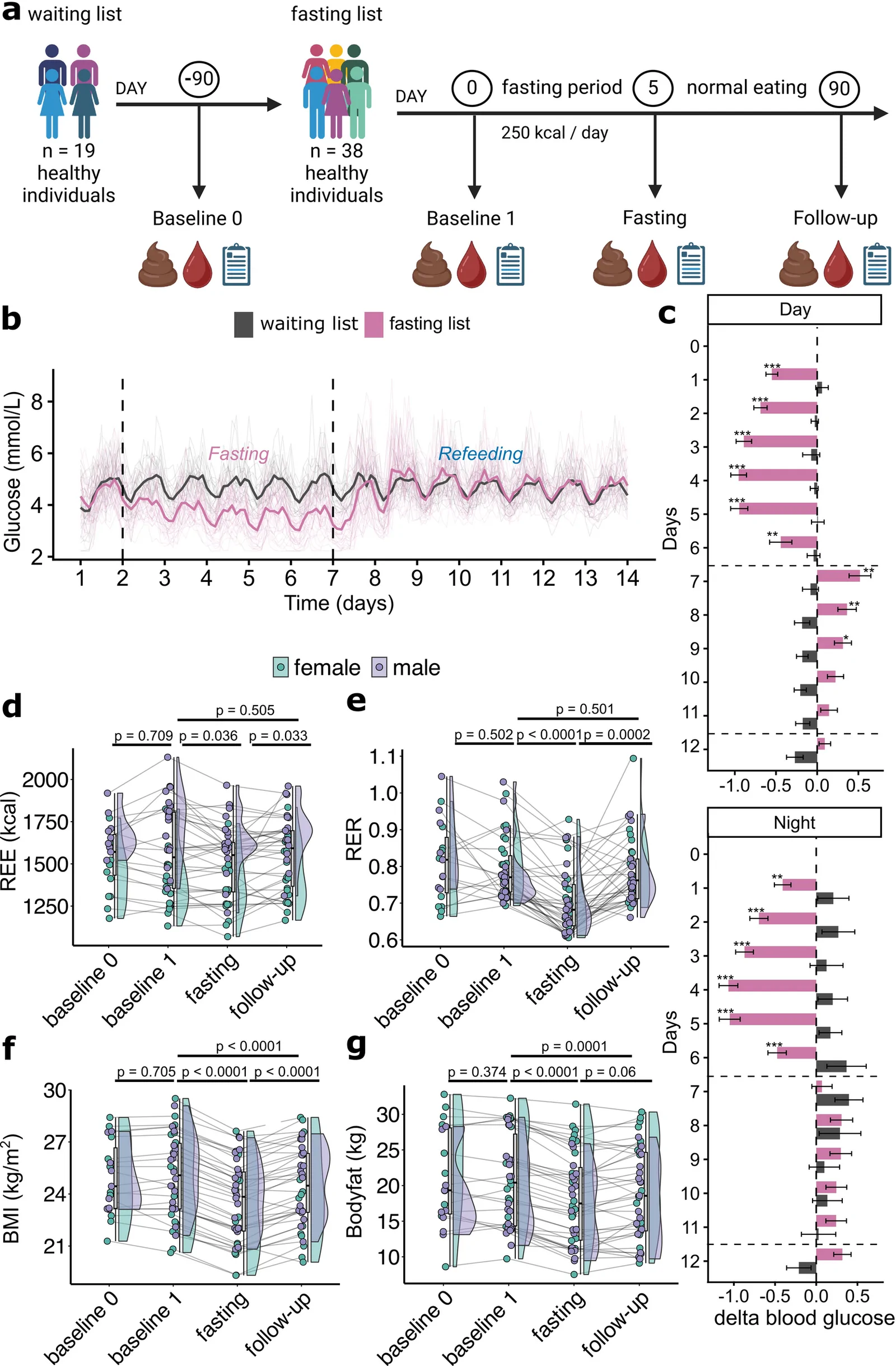
Physiological effects of prolonged fasting in healthy individuals. **a**) Schematic overview of the randomized waitlist-controlled trial. A total of 38 healthy participants were recruited. The first 19 participants immediately underwent prolonged fasting, while the remaining 19 served as a waitlist control group. After the initial intervention period, the waitlist group also underwent the same prolonged fasting protocol, resulting in a complete dataset of 38 participants undergoing prolonged fasting. The fasting intervention consisted of a 5-day period with 250 kcal/day, preceded and followed by baseline and follow-up visits at days 0, 5, and 90. Stool, blood, and clinical data were collected at each visit. The waitlist group was assessed at an additional baseline (day − 90) to control for temporal fluctuations. **b**) Glucose time series measured by continuous intradermal monitoring starting one day prior to fasting (pink) or at baseline for the waiting list control (grey). **c**) Quantification of absolute changes in mmol/L of mean daytime (6am to 10pm) (top) and night (10pm to 6am) glucose levels (bottom), with changes compared to the individual baseline (day 1). Paired t-test with Benjamini-Hochberg false discovery rate correction (BH-FDR), *q < 0.1, **q < 0.01, ***q < 0.001. **d**) Resting energy expenditure (REE), **e**) respiratory exchange ratio (RER), **f**) body mass index (BMI), and **g**) body fat percentage at the different study visits. Each point represents one participant, lines connect individuals, boxplot (middle) for all participants, violins (right side) and dots coloured by sex. P-values are from Wilcoxon signed-rank tests and were corrected using the Holm method

We performed a randomized, waitlist-controlled study (LEANER study, ClinicalTrials.gov; NCT04452916, submitted 29.06.20290) carried out at the Experimental and Clinical Research Center of Charité – Universitätsmedizin in Berlin, from 02 November 2020 (enrolment of the first patient) to 14 December 2022 (primary completion date). The study protocol (Additional file 1) was approved by the institutional review board of Charité – Universitätsmedizin Berlin (EA1/033/20) and all participants provided written informed consent before entering the study. We collected and managed study data using REDCap electronic data capture tools hosted at Charité – Universitätsmedizin Berlin [22, 23]. This study was exploratory, and we did not calculate a pre-determined sample size. Instead, the size of the recruited cohort was determined based on methodological and feasibility considerations. Flow diagram of recruitment and enrollment of patients according to Consort 2025 guidelines [24] is shown in Additional file 2: Figure S1. A general overview of the study is provided in Fig. 1a.

### Eligibility criteria

We included individuals aged 20–50 years who were assigned male or female at birth and identified as men and women, respectively; with a body mass index between 20 and 30 kg/m^2^. Exclusion criteria were heart, lung, liver, and kidney diseases requiring medical intervention, prescribed medication (except oral contraceptives), postoperative phases, current or chronic infection, antibiotic treatment or a fasting week within the last 6 months, habitual use of dietary supplements, food intolerances, > 2 kg body weight change within the last month, pregnancy, lactation, vegan diet, smoking, drug, or alcohol abuse.

### Intervention

Participants received oral and written instructions on the method of prolonged fasting. Expert guidance was provided by experienced nutritionists and certified fasting counselors in a group setting with 5–10 participants per group. After sufficient instruction, the fasting was performed individually at home, with the possibility of reaching out to the counselor at any time or to connect with other group members (if patients gave consent to share this information). Participants were encouraged but not required to take time off during this week. Fasting was preceded by one preparation day, on which participants consumed a low-calorie, fiber-rich diet, e.g. fruits, vegetables, potatoes, rice, and oats.

During the five fasting days, participants were allowed to consume 200–250 kcal/d through vegetable juices and vegetable broths. Herbal teas and water could be consumed ad libitum. At the morning of fasting day 6, we performed the study visit and patients were instructed for the refeeding phase. A detailed fasting plan including refeeding recommendation is provided as Additional file 2: Supplementary Table S1.

### Randomization

We assigned participants randomly according to a locked list created by www.randomizer.at (two treatments: waiting, fasting; 1 factor: self-reported sex; permuted blocks; block sizes 4, 2, 6, 4). Due to the nature of the intervention and the wait-list design, blinding was not possible.

### Outcome measures

We established all outcome measures in advance and did not adapt them during or after the study. The primary outcome measure was resting energy expenditure (REE in kcal/d) after 5 days of fasting vs. baseline 1 assessed by indirect calorimetry. The primary outcome was analyzed immediately after study completion and the primary endpoint was met. Subsequently, secondary outcomes were analyzed. Secondary outcomes were body composition, cardiovascular parameters, various routine blood parameters, glucose variability, gut metagenome, fecal, and plasma metabolome. In addition, we assessed compliance and safety of fasting. Key secondary analyses include multivariate and univariate analysis of each space as well as subsequent predictive machine-learning based analysis.

### Study center assessments

The waiting group underwent assessments four times, the fasting group three times. The fasting group had one baseline visit (baseline 1) and started the intervention within two to eleven days afterwards, the waiting group had two baseline visits with a 12-week waiting period in between (baseline 0 and baseline 1) and also started the intervention within a maximum of 11 days after baseline 1. These two baselines allowed detection of random time effects on the assessed outcome measures. For both groups, the next visit was on the morning of the sixth fasting day directly before breaking the fast (fasting). To assess medium-term effects of fasting, there was another visit after 12 weeks (follow-up). Except for the additional baseline 0 in the waiting group, all assessments were identical between the groups. All examinations described hereafter were performed at all study visits.

Assessments commenced in the morning following a 12-hour overnight fast. Participants were instructed to abstain from caffeine, alcohol, and vigorous exercise for 24 h prior to the assessments.

Venous blood was drawn and sent to an accredited laboratory (Labor Berlin, Berlin, Germany) that measured parameters of glucose and lipid metabolism, blood count, electrolytes, liver enzymes, uric acid, CRP, and renal function according to international standards.

We measured waist and hip circumference to calculate waist-to-hip ratio and determined body composition (lean and fat mass) by air displacement plethysmography (Bod Pod, Life Measurements, Inc., Concord, CA).

Indirect calorimetry was conducted following a 30 min resting period while in a supine position within a thermoneutral environment for a duration of 30 min (Quark RMR, COSMED, Italy). REE and respiratory exchange ratio (RER) were calculated from oxygen consumption and carbon dioxide production as described elsewhere [25]. 

Office blood pressure and heart rate were determined by five automated measurements within 10 min with an upper arm cuff (Mobil-o-Graph, I.E.M. GmbH, Stolberg, Germany) in the absence of study personnel.

### Independent cohorts

To assess the transferability of the BMI-response classifier, we re-analyzed clinical and shotgun metagenomic data from three previously published prolonged-fasting cohorts [7, 26, 27]. The Maifeld cohort comprised 34 participants with metabolic syndrome who underwent either a 5-day prolonged fasting intervention followed by a DASH diet or a DASH diet alone (ClinicalTrials.gov: NCT02099968), with follow-up after 12 weeks [7]. The Bahr cohort comprised 22 patients with multiple sclerosis who underwent two 7-day prolonged fasting periods interspersed with intermittent fasting (ClinicalTrials.gov: NCT03508414), with assessments performed nine months apart [26]. The Ducarmon dataset comprised healthy participants from the GENESIS (ClinicalTrials.gov: NCT05449249) and OralFast (ClinicalTrials.gov: NCT05031598) studies who underwent 6–12 days of prolonged fasting [27]. For the present analysis, clinical and metagenomic data from 47 participants from these two subcohorts were used, with outcome assessment after approximately 4 months in GENESIS and 3 months in OralFast. Detailed recruitment procedures, eligibility criteria, sample collection protocols, and original study procedures for all external cohorts have been described in the respective primary publications [7, 26, 27].

For the present study, sequencing data from all external cohorts were reprocessed using the same microbiome-processing pipeline as applied to the LEANER cohort, as described below. The trained LEANER classifier was subsequently applied without retraining to the Maifeld, Bahr, and Ducarmon datasets to assess its transferability across independent fasting cohorts. Cohort-specific responder definitions were based on the available control or follow-up structure of the respective studies. Model discrimination and calibration were assessed where permitted by the respective study design and available outcome information; post-hoc calibration analyses were performed for the Maifeld and Ducarmon cohorts. Cohort-specific exclusions resulting from microbiome sequencing-depth requirements are detailed in the Microbiome data processing subsection below.

### Fecal samples

At each study visit, participants received two stool collection kits and were asked to collect two samples from one stool at home as soon as possible following the study visit. One sample was used for gut metagenome sequencing (OMNIgene Gut, DNA Genotek, Ottawa, Canada) and the other for metabolome analysis (OMNImet Gut, DNA Genotek, Ottawa, Canada). These kits allow shipment without cooling to the study center. Participants sent the tubes to the study center at room temperature, where they were stored at − 80 °C until further processing.

### Glucose sensors

We assessed glucose variability by continuous glucose monitoring over 14 days. For this, participants wore a glucose sensor that measured interstitial glucose concentrations in the subcutaneous tissue of the dorsal upper arm every 15 min (FreeStyle Libre Pro, Abbott, Chicago, IL, USA). In contrast to the use of these sensors in diabetic patients, participants in this study could not see their values during the measurement. Baseline assessment was done only in the waiting group directly following baseline visit 0. Additionally, fasting assessment was done in all participants during preparation, fasting, and refeeding.

### Ketone and glucose measurements

On the five fasting days, participants independently measured their blood concentration of beta-hydroxybutyrate (BHB) and glucose twice daily using a handheld glucometer (Freestyle Precision Neo, Abbott, Chicago, IL, USA), and recorded their values in a provided protocol. In addition, values were stored on the device and read out later in the study center, providing an objective measure of compliance.

### Fecal metagenomic sequencing

#### DNA isolation

DNA was isolated using ZymoBIOMICS DNA Miniprep Kit (ZymoReseach Europe GmbH, Freiburg, Germany). In short, an aliquot of 250 µL of stool sample from the OMNIgene Gut tube was added to a ZR BashingBead Lysis Tube. The following extraction was done according to the manufacturer’s instructions. DNA was eluted in 100 µL RNase/DNase-free water and collected in a 1.5-mL Eppendorf tube. Total DNA concentration was measured using NanoDrop ND-1000 (Peqlab Biotechnologie GmbH, Erlangen, Germany). Isolated DNA was stored at -80 °C [28].

#### Shotgun metagenomics

Isolated DNA was shipped on dry ice to Novogene (Cambridge, UK) for shotgun metagenomic DNA sequencing. In short, DNA was fragmented and subsequently underwent end repair and phosphorylation. After A-tailing, adapter ligation was performed. Then 150 bp paired-end sequencing was performed on the Illumina Novaseq 6000 platform with an aimed data collection of 6Gb raw data.

#### Microbiome data processing

Sequencing reads were mapped to the mOTUv2 (version 2.6) database, and read counts were subsequently rarefied using the RTK R package (version 0.2.6.1) to standardize sequencing depth across samples. Rarefaction involves randomly subsampling reads to match the sample with the lowest read depth, thereby mitigating biases introduced by variable sequencing depth and enabling reliable comparisons across cohorts. For the LEANER (healthy) cohort, a cutoff of 3,859 reads was applied, resulting in the exclusion of one sample due to insufficient sequencing depth. The same threshold was used for the Maifeld cohort [7], where one sample was also excluded. In the Bahr dataset [26], applying the same rarefaction criteria led to the exclusion of one sample. In the Ducarmon dataset [27], applying the consistent rarefaction criteria resulted in the exclusion of 9 baseline samples (7 from GENESIS and 2 from OralFast trial). This consistent approach enabled reliable comparisons across cohorts and increased the robustness of downstream analyses.

#### Metagenome assembly

To recover representative genomes of the dominant *Oscillibacter* and *Faecalibacterium* lineages, shotgun metagenomic reads from the selected samples were pooled prior to assembly. Because the combined paired-end dataset exceeded the memory available for a single assembly (> 280 GB compressed FASTQ), reads were partitioned into nine paired subsets using BBTools reformat.sh v39.19, while preserving read pairing. Each partition contained approximately 50 million paired reads and was assembled independently using MEGAHIT v1.2.9). Contigs generated from all partitions were subsequently merged into a single assembly for downstream analyses.

#### Genome binning

Merged contigs were grouped into metagenome-assembled genomes (MAGs) using MetaBAT2. Contig coverage was estimated by mapping the original reads back to the assembled contigs. Genome completeness and contamination were evaluated using CheckM. Candidate MAGs were ranked according to estimated completeness and contamination, and the highest-quality bins corresponding to the target taxa were selected for downstream analyses. Taxonomic classification of all MAGs was performed using GTDB-Tk v2.4.0 with GTDB release R220. Selected MAGs were annotated using Prokka. Metabolic reconstruction was performed using DRAM. DRAM assigns KEGG Orthology identifiers, metabolic modules and pathway annotations from predicted protein sequences. Presence or absence of genes involved in butyrate biosynthesis, tryptophan/indole metabolism and other pathways of interest was determined from the DRAM annotation outputs. Gene presence in each MAG was summarized manually and visualized as a presence/absence matrix.

### Plasma metabolomics

#### Collection of plasma

Venous blood was collected in Vacutainer^®^ EDTA tubes (BD, Heidelberg, Germany) and immediately put on crushed ice for 30 min and then centrifuged (3,000 x g, 4 °C, 10 min). Plasma was collected and stored at − 80 °C.

#### Preparation of plasma for nuclear magnetic resonance spectroscopy (NMR)

Plasma samples were prepared with a robotic system (SamplePro Tube, Bruker BioSpin GmbH, Ettlingen, Germany) by mixing 287 µL plasma with 287 µL IVDr buffer (Bruker BioSpin GmbH, Ettlingen, Germany) and adding 10 µL formic acid (240 mM) as internal standard.

#### NMR measurement of plasma

All NMR experiments were performed on a 600 MHz Bruker Avance III spectrometer, using a triple resonance (^1^H, ^13^C, ^15^N, ^2^H lock) helium cooled cryoprobe with z-gradient. Samples were handled by an automatic Bruker SampleJet sample changer (Bruker Biospin GmbH, Ettlingen, Germany). Tuning and matching of the probe as well as locking and shimming of the sample were performed automatically. Following the Bruker IVDr protocol for plasma measurements for each sample, four different types of ^1^H NMR spectra were acquired at 310 K (1D NOESY, 2D JRES, 1D CPMG, and 1D spin echo diffusion spectrum).

#### Data analysis of plasma NMR spectra

From the collected spectra of each specimen, 41 smaller, non-lipoprotein metabolites were automatically identified and quantified using the Bruker IVDr Quantification in Plasma/Serum B.I.Quant-PSTM platform. Of note, only metabolites levels not bound to proteins were determined. Additionally, 112 lipoprotein parameters including various lipoprotein fractions, classes and subfractions were identified and quantified using the Bruker IVDr Lipoprotein Subclass Analysis B.I.LISATM platform.

### Stool metabolomics

#### Drying of stool samples

Fecal samples from OMNImet Gut tubes were processed for metabolomics. Fecal samples (1 mL) were dissociated two times after adding 2.5 mL 70% isopropanol using C tubes with the gentleMACS OctoDissociator (Miltenyi Biotec, Bergisch Gladbach, Germany). Aliquots of dissociated samples were covered with perforated Parafilm and dried overnight in a vacuum concentrator (Concentrator 5301, Eppendorf, Hamburg, Germany). Dried samples were stored at − 80 °C.

#### Preparation of stool for NMR

Dried stool samples were resuspended in 600 µL (< 10 mg dry weight), 800 µL (10–40 mg dry weight) or 1,000 µL (> 40 mg dry weight) of double distilled water and 10 µL of an extraction standard (Nicotinic acid at 80 mmol/L) were added. Samples were centrifuged three times (12 000 x g, 4 °C, 10 min) and supernatants were transferred into a fresh vial after each centrifugation to remove particles. Of the final supernatant, 400 µL were mixed with 200 µL of 0.1 mol/L phosphate buffer (pH 7.4) and 50 µL of a 0.75% (w/v) solution of 3-trimethylsilyl-2,2,3,3-tetradeuteropropionate (TSP; Sigma-Aldrich, Taufkirchen, Germany), which served as an internal standard, in deuterium oxide.

#### NMR measurements of stool

NMR experiments were conducted on the same NMR spectrometer as described for the plasma analyses. Before measurement, each sample was allowed to equilibrate for 300 s in the magnet, and the probe was automatically locked, tuned, matched, and shimmed. One-dimensional ^1^H NMR spectra were obtained at 298 K using a nuclear Overhauser enhancement spectroscopy pulse sequence with solvent signal suppression by pre-saturation during relaxation and mixing time. For each spectrum, 512 scans were collected into 65,536 data points over a 20-ppm (parts/million) spectral width using a relaxation delay of 4 s, an acquisition time of 2.66 s, and a mixing time of 0.01 s. Spectra were automatically Fourier transformed and phase and baseline corrected.

#### Data analysis of stool NMR spectra

In each spectrum, a set of 41 metabolites were semi-automatically identified and relative to the TSP reference signal quantified using the CHENOMX 9.02 (Chenomx Inc, Edmonton, Canada) software suite. To account for potential losses during sample preparation, resulting data was adjusted to the extraction standard and normalized to dry weight.

### Predictive modelling of BMI response

To predict sustained BMI response to prolonged fasting, we used a random forest classifier implemented in the caret package (R 7.0.1). The outcome variable was responder status (responder vs. non-responder) defined by a BMI reduction exceeding the mean plus two standard deviations of the waiting list control group. Given the class imbalance (38% responders and 62% non-responders), stratified Monte-Carlo resampling was used to preserve the class proportions in each training and test split. Predictor variables included either (i) the full set of 87 microbiome and clinical features (Supplementary Data 6) or (ii) a predefined sparse model with four predictors: *Oscillibacter sp.* 57_20, *Faecalibacterium sp.* incertae sedis, systolic blood pressure, and LDL cholesterol. Given the limited sample size, we used 100 stratified 80/20 Monte-Carlo resamples with inner 5-fold cross-validation for hyperparameter tuning, using the area under the ROC curve (AUC) as the optimization metric. The best-performing model from each resample was evaluated on its holdout 20% test partition, providing a distribution of performance estimates rather than a single value.

Model performance was assessed by AUC, accuracy, sensitivity, specificity, balanced accuracy, F1 score, Matthews correlation coefficient (MCC), and average precision (area under the precision–recall curve). We report both mean ± SD and median [IQR] across resamples to capture variability. Feature importance was obtained via permutation importance, and stability summarized as mean importance ± SD and rank distribution. Feature-stability analysis showed *Oscillibacter sp.* 57_20 and *Faecalibacterium sp.* incertae sedis consistently ranked highest, systolic BP contributed modestly, and LDL showed low and variable importance. LDL was retained in the predefined four-feature model to avoid post-hoc selection, while a three-feature variant performed comparably (Supplementary Table S3).

To assess potential confounding and the added predictive value of microbiome data, we trained two additional models using the same resampling procedure: (i) a clinical-only model (age, sex, baseline BMI, blood pressure, lipid parameters), and (ii) a combined model incorporating microbiome predictors.

### Calibration

To evaluate the reliability of predicted probabilities, we applied post-hoc calibration using Platt scaling (logistic correction of the logit scores) and isotonic regression (monotone, non-parametric mapping) in the Maifeld and Ducarmon cohort. The original model was not retrained. Instead, we performed 100 stratified 80/20 Monte-Carlo resamples, ensuring that both outcome classes were present in each training and test set. For every resample, the calibration models were fit on the training fold only and then applied to the hold-out test fold. We compared three variants of prediction: the raw model (no calibration), Platt-scaled, and isotonic-scaled probabilities.

On each test fold, we calculated AUC, accuracy, sensitivity, specificity, balanced accuracy, F1 score, Matthews correlation coefficient (MCC), and the Brier score (mean squared error of predicted probabilities). Predictions were dichotomized at a 0.5 threshold to form confusion matrices, summarized as mean ± SD of the test proportion. Calibration was further assessed using Calibration-in-the-Large (CITL) and Calibration Slope in the test fold. All performance measures were summarized as mean ± SD across the 100 repeats.

### Statistical analysis

Descriptive statistics for interval variables are presented as mean ± standard deviation or median with interquartile range (IQR). For comparisons of delta BMI between responders and non-responders, normality of variable distributions was assessed by visual check of histograms and qq-plots. Mann-Whitney U-test or t-test was used as appropriate. For all other data, comparisons between independent groups were performed using the Mann-Whitney U test, and paired samples were compared using the Wilcoxon signed-rank test. Where applicable, p-values were adjusted for multiple comparisons using either the Holm method (for family-wise error control) or the Benjamini-Hochberg (BH) procedure (for false discovery rate control), depending on the analytical objective.

Permutational Multivariate Analysis of Variance (PERMANOVA) was used to test for multivariate compositional differences between groups and visits.

To evaluate the relationship between bacterial abundance and metabolite concentrations while accounting for visit timepoint, we performed permutation-based conditional independence tests. Specifically, for each metabolite, its association with *Oscillibacter* sp. 57_20 and *Faecalibacterium* sp. incertae sedis was tested while conditioning on the visit variable, using the R package coin (version 1.4.3) with 10,000 permutations. Resulting p-values were corrected for multiple testing using the Benjamini-Hochberg false discovery rate (FDR) method.

Statistical significance was defined as *p* < 0.05 or q < 0.1, where applicable.

## Results

### Prolonged fasting affects energy expenditure and weight loss at the 12-week follow-up

Thirty-eight healthy participants were recruited for a 5-day prolonged fasting intervention (Fig. 1a). As fasting interventions cannot be blinded, we decided to use a waiting list control to account for physiological fluctuations within a 12-week timeframe while maximizing the number of participants undergoing prolonged fasting (Fig. 1a). Baseline characteristics are shown in Table 1.

**Table 1:** Baseline characteristics of healthy participants depending on their randomization to fasting and waiting control arm

Variable	All	Fasting	Waiting	*P* value
Number (n)	38	19	19	
Sex, female	19 / 38 (50%)	9 / 19 (47%)	10 / 19 (53%)	> 0.9
Age (years)	38 (8)	39 (9)	36 (8)	0.4
BMI (kg/m^2^)	25.1 (2.5)	25.0 (2.7)	25.2 (2.3)	0.7
eGFR (ml/min/1.73m^2^)	102 (14)	98 (12)	106 (15)	0.1
ALT (U/l)	23.1 (8.1)	23.0 (8.2)	23.2 (8.2)	0.2
TSH (mU/l)	1.70 (0.84)	1.77 (0.91)	1.64 (0.77)	0.6
CRP (mg/dl)	0.98 (0.87)	0.78 (0.28)	1.17 (1.17)	0.2
Cholesterol (mg/dl)	182 (36)	189 (35)	175 (36)	0.2
HbA1c (mmol/mol)	33.2 (2.8)	34.2 (2.6)	32.3 (2.7)	0.03
Hemoglobin (g/dl)	14.0 (1.3)	13.8 (1.3)	14.2 (1.2)	0.41
White blood count (uL^− 1^)	5.46 (1.02)	5.56 (0.96)	5.37 (1.09)	0.6
Thrombocytes (uL^− 1^)	240 (42)	238 (63)	239 (54)	0.9

Data are given as means (SD). p-values fasting vs. waiting by t-test. BMI, body mass index; eGFR, estimated glomerular filtration rate; ALT, alanine aminotransferase; TSH, thyroid-stimulating hormone; CRP, C-reactive protein; HbA1c, glycated hemoglobin

Of the 38 participants, 83% reported fasting-related adverse events (AE) (Additional file 2: Table S2). All AE were mild and transient and did not necessitate discontinuation of fasting. Blood glucose and β-hydroxybutyrate (BHB) levels were measured each morning and evening during the five-day fasting period (days 1–5), confirming that all participants adhered closely to the study protocol (Additional file 2: Figure S2a, b). We observed a diurnal rhythm with higher BHB but lower glucose concentrations in the morning compared to the evening (Additional file 2: Figure S2a, b). Continuous interstitial glucose monitoring during the five-day fasting period, compared with baseline measurements and waiting-list controls, confirmed a dynamic reduction in glucose levels over the course of the fasting period (Fig. 1b). Following refeeding, the glucose levels normalized; however, between days 2 and 4 post-fast, blood glucose concentrations were elevated throughout the day (06:00–22:00) compared with baseline measurements (Fig. 1c).

Our predefined endpoint was a change in REE. Prolonged fasting acutely reduced REE by 62 kcal/d, suggesting a fasting-induced, energy-preserving mechanism (Fig. 1d, Additional file 3). We did not observe alterations in REE between the two baseline timepoints of waiting list controls (Fig. 1d). Following the findings of earlier work [12], we performed a linear mixed model showing that lean mass (*p* < 0.001) and not self-reported sex significantly influenced REE at all visits, including post-fasting (Additional file 2: Figure S3). Following the five-day fast, respiratory exchange ratios (RER) declined significantly (Fig. 1e), paralleling a peak in BHB concentrations on day 5 (Additional file 2: Figure S2b). All those metabolic markers returned to baseline by 12 weeks post-fast.

Furthermore, we observed that prolonged fasting reduced BMI (Fig. 1f). This decrease was partly reversed at the 12-week follow-up (Fig. 1f). Body composition analysis attributed this incomplete return towards baseline BMI predominantly to a reduction in fat mass (Fig. 1g, Additional file 2: Figure S2g). Of note, the absolute and relative reduction of body fat was accompanied by a reduction in lean mass during fasting that completely reverted at follow-up (Additional file 2: Figure S2h).

Prolonged fasting reduced waist-to-hip ratio and diastolic blood pressure while increasing heart rate, findings which are consistent with a previously published study [7] in metabolic syndrome (Additional file 2: Figure S2c-e). In contrast to metabolic syndrome patients [7], the healthy participants in this study did not exhibit sustained blood-pressure reduction (Additional file 2: Figure S2d-e).

Taken together, these results demonstrate that prolonged fasting is well tolerated in healthy individuals, yielding sustained reductions in BMI and fat mass, while most metabolic and hemodynamic parameters return to baseline during the 12-week follow-up.

### Prolonged fasting induces pronounced short-term but only modest sustained changes in gut microbiome composition

**Fig. 2:**
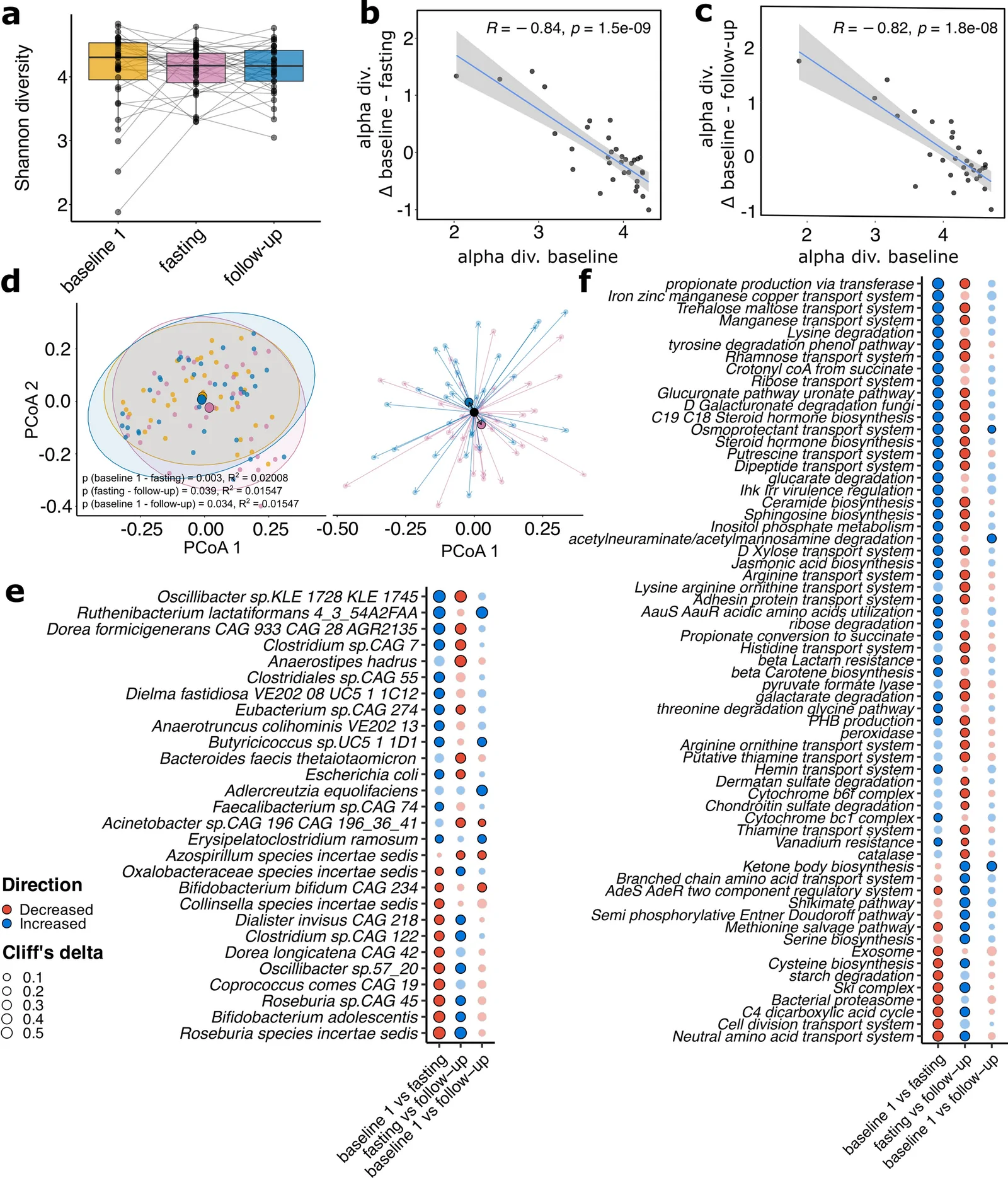
Prolonged fasting induces transient alterations in gut microbiome composition and function. (**a**) Alpha diversity as measured by Shannon index for the study visits. Correlation of Shannon index at baseline with changes in Shannon index immediately (**b**) post-fasting and (**c**) at 12-week follow-up, indicating that baseline diversity predicts microbiome responsiveness. (**d**) Principal coordinate analysis (PCoA) based on Bray–Curtis dissimilarity (naïve, left) and centered on baseline visit for fasting effect and on fasting visit for post-fasting effect (right) show a transient shift in overall microbial composition after fasting (pink), which partially reverts at follow-up (blue). Ellipses represent 95% confidence intervals, p-values from PERMANOVA. Dot plots showing differential abundant (**e**) species (taxonomic level) and (**f**) gut metabolic modules (functional level). Dots show fasting effects, follow-up effects and study effects, transparency indicates non-significant findings (q > 0.1), dot size shows absolute Cliff’s delta, color shows directionality. Significance by Wilcoxon signed-rank test with Benjamini–Hochberg false discovery rate correction

To assess the natural fluctuation of the gut microbiome composition, we analyzed changes between the two baseline samples (baseline 0 and baseline 1) from the waiting list control (Fig. 1a). Microbial alpha diversity, measured as Shannon index, and microbiome composition quantified by Bray-Curtis dissimilarity did not differ significantly between the two baseline samples (Additional file 2: Figure S4a, b). Shannon index remained stable across all study visits (Fig. 2a). In contrast, individual trajectories in response to fasting varied markedly (Fig. 2a). Interestingly, short-term (post-fasting) and 12-week follow-up changes in alpha-diversity correlated negatively with alpha-diversity at baseline, implicating baseline differences in microbiome composition as relevant predictors of fasting-induced microbiome changes (Fig. 2b, c). Bray–Curtis dissimilarity-based multivariate analysis of the taxonomic composition demonstrated a significant shift in microbial community composition from baseline to fasting (PERMANOVA, *R*² = 0.0201), followed by a partial reversal during the 12-week follow-up (*p* = 0.039, *R*² = 0.0155). Despite this reversal, microbial community composition at the 12-week follow-up remained significantly different from baseline (*p* = 0.034, *R*² = 0.0155) (Fig. 2d). Consistent with the observed shift in the multivariate analysis, fasting induced significant changes in several bacterial species that reversed during follow-up. For example, *Roseburia sp.* inserta sedis and *Bifidobacterium adolescentis* decreased after the fasting period but increased at follow-up, whereas *Faecalibacterium sp.* CAG-74 showed the opposite pattern (Fig. 2e, Additional file 2: Figure S4c, Additional file 4). In contrast, only seven taxa exhibited sustained abundance changes (Additional file 2: Figure S4c).

Next, we analyzed gut-specific metabolic modules using GOmixer [29]. Again, fasting induced pronounced alterations that largely reverted to baseline during follow-up (Fig. 2f). Only for three modules, ketone body synthesis, osmoprotectant transport system, and N-acetylneuraminate and N-acetylmannosamine degradation, fasting induced a long-lasting increase when comparing baseline to follow-up (Fig. 2f, Additional file 2: Figure S5a, Additional file 5).

Overall, prolonged fasting induces pronounced compositional shifts in the gut microbiome, which were largely reversed upon refeeding, resulting in minimal long-term deviations from baseline at the 12-week follow-up. Notably, baseline alpha diversity metrics correlate with the magnitude of alpha diversity changes both immediately after fasting and at follow-up.

#### Prolonged fasting alters plasma and fecal metabolome

We performed nuclear magnetic resonance (NMR) spectroscopy on plasma and fecal samples to assess the impact of fasting, and its associated microbiome shifts on the fecal and host metabolome. Principal coordinate analysis (PCA), centered on baseline values to characterize post-fast changes and on post-fast values to characterize follow-up effects, revealed plasma metabolome trajectories analogous to those observed in the gut microbiome (Additional file 2: Figure S5b). In line, we observed significant alterations between baseline, fasting, and follow-up, with most changes reversed at follow-up (Additional file 2: Figure S5c, Additional file 6). In plasma, only valine, glycine, tyrosine, and pyruvic acid exhibited sustained increases from baseline to follow-up, indicating lasting effects of fasting on these metabolites (Additional file 2: Figure S5b, c).

Fecal metabolite profiling again revealed a pattern of alteration and reversion to baseline (Additional file 2: Figure S5d). Contradicting other previous reports on short-chain fatty acids (SCFA) in fasting [7], we observed a decrease in butyrate during fasting, which was non-significant at follow-up (Additional file 2: Figure S5e, Additional file 7). The only persistent effect observed in our data was a decrease in choline from baseline to fasting (Additional file 2: Figure S5e).

In conclusion, NMR-based metabolomics of plasma shows more consistent alterations as compared to the fecal metabolome, which might be due to the different matrix or intraindividual variations in gut microbiome composition.

#### Prediction of sustained BMI response by multi-omics machine learning

BMI showed the strongest sustained effect at the 12-week follow-up among the analyzed clinical parameters (Fig. 1). Therefore, we focused on the weight-loss response at the 12-week follow-up as the primary phenotype to predict. Using the waiting-list control, we estimated the normal fluctuation in BMI over the 12-week study period. Normal fluctuation in BMI was defined as the mean ± two standard deviations (SD) [30], corresponding to − 0.008 ± 0.32 kg/m² in our cohort. Based on this threshold, we identified 38% responders and 62% non-responders to prolonged fasting.

**Fig. 3:**
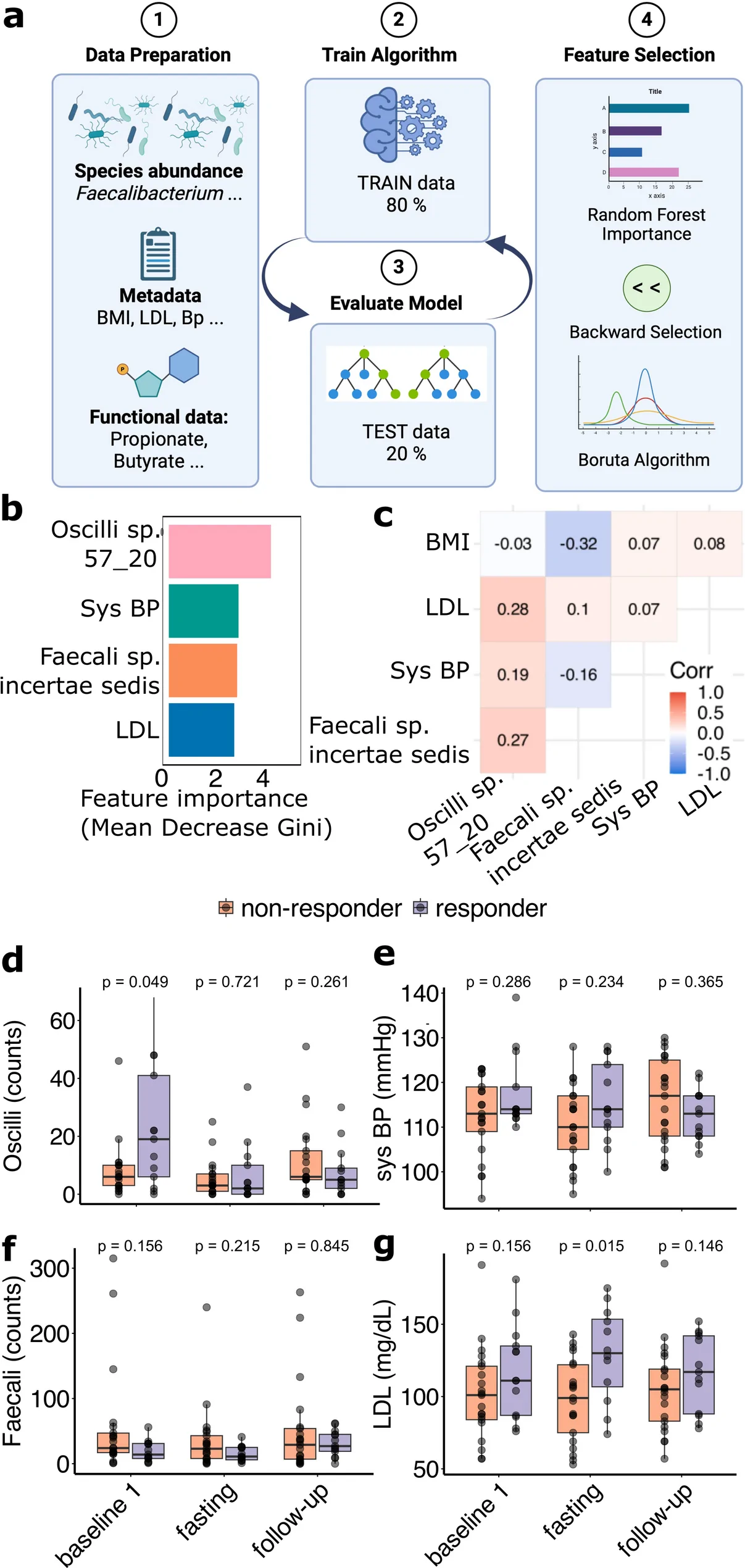
Machine learning model predicts sustained BMI response at follow-up to prolonged fasting using clinical and microbiome features. (**a**) Schematic overview of the multi*-omics* feature selection and classification pipeline. Baseline species abundances, clinical metadata, and functional modules were used to train a random forest classifier. Important features were selected through recursive feature elimination, Boruta algorithm, and backward selection. (**b**) Final model includes four top-ranked features: *Faecalibacterium sp.* incertae sedis, *Oscillibacter sp.* 57_20, LDL cholesterol, and systolic blood pressure. (**c**) Correlation heatmap of the selected features showing independence from baseline BMI. R-values from Spearman correlation. (**d**) Boxplots of the four selected features at baseline, comparing BMI responders and non-responders, dots show individual patients. P-values by Mann-Whitney-U-test. Oscilli, *Oscillibacter sp.* 57_20; Faecali, *Faecalibacterium sp.* incertae sedis; LDL, Low-density lipoprotein cholesterol; sys BP, systolic blood pressure

To predict BMI loss response, a random forest classifier was trained on 87 features including the 50 most abundant microbial species at baseline, additional candidate taxa and pathways from previous prolonged fasting studies, and relevant clinical metadata (Additional file 8). To obtain a model that would be as transferable as possible to publicly available datasets, we focused on microbiome and clinical features rather than NMR metabolomics [7, 26]. Due to the small sample size, we applied a repeated cross-validation approach to ensure robust model evaluation. Specifically, we used 100 repeated stratified 80/20 resamples, each incorporating an inner 5-fold cross-validation loop for hyperparameter tuning. Model performance was further optimized by performing feature selection once after model tuning, based on random-forest feature importance, the Boruta algorithm, and backward feature elimination (Fig. 3b, Additional file 2: Figure S6c). This resulted in four features: LDL, systolic blood pressure (BP), *Faecalibacterium sp.* incerta sedis and *Oscillibacter sp.* 57_20. Those four features were used to train the final model. Across the repeated 80/20 resampling, the refined short model performed well with an AUC of 0.84 ± 0.17 (Table 2).

**Table 2:** Summary of predictive performance metrics for the predefined sparse 4-feature model, evaluated using 100 iterations of stratified 80/20 Monte Carlo resampling

Metric	Value (mean ± SD)
AUC	0.84 ± 0.17
Accuracy	0.76 ± 0.15
Sensitivity	0.56 ± 0.34
Specificity	0.85 ± 0.17
Balanced Accuracy	0.71 ± 0.18
F1	0.68 ± 0.18
MCC	0.50 ± 0.35

Values are reported as mean ± SD across resamples. AUC, area under the receiver operating characteristic curve; F1, F1 score (harmonic mean of precision and recall); MCC, Matthews correlation coefficient; SD, standard deviation

Baseline BMI can confound weight loss prediction. Therefore, we tested collinearity between baseline BMI and our four selected features, none of which reached significance (Fig. 3c). To test if the four selected features are mere surrogate markers of baseline BMI, we incorporated baseline BMI into the model. This did not improve predictive accuracy (Additional file 2: Figure S6a, b). Furthermore, *Oscillibacter sp.* 57_20 remained the most important feature. Feature-stability analysis supported this ranking: *Oscillibacter sp.* 57_20 and *Faecalibacterium sp.* incertae sedis consistently ranked highest, followed by systolic BP, while LDL showed low and inconsistent importance (− 0.9 ± 3.2; Additional file 2: Supplementary Table S3). To avoid post-hoc feature removal, LDL was retained in the sparse model, although a 3-feature variant performed similarly (Additional file 2: Supplementary Table S4). Next, we compared a clinical-only model (including baseline BMI, blood pressure, and lipid parameters) with a combined model that additionally incorporated microbiome data to address potential confounding by clinical features (Table 3). The clinical-only model achieved an AUC = 0.69 ± 0.21, accuracy = 0.66 ± 0.18, and balanced accuracy = 0.62 ± 0.19 (Table 3). The combined model improved performance across all metrics (AUC = 0.86 ± 0.17, accuracy = 0.74 ± 0.16, balanced accuracy = 0.72 ± 0.18, Table 3). These results indicate that microbiome features contribute predictive information beyond standard clinical covariates, underscoring their added value for identifying responders to prolonged fasting.

**Table 3:** Comparison of predictive performance of clinical-only versus clinical+microbiome models

Metric	Clinical-only model	Clinical + Microbiome model
AUC	0.69 ± 0.21	0.86 ± 0.17
Accuracy	0.66 ± 0.18	0.74 ± 0.16
Sensitivity	0.52 ± 0.34	0.66 ± 0.35
Specificity	0.73 ± 0.22	0.78 ± 0.19
Balanced Accuracy	0.62 ± 0.19	0.72 ± 0.18
F1	0.60 ± 0.18	0.69 ± 0.16
MCC	0.28 ± 0.41	0.48 ± 0.37

Predictive performance of models trained on clinical features alone compared with models including both clinical and microbiome features. Values represent mean ± SD across 100 repeated stratified 80/20 resamples. Inclusion of microbiome data improved classification metrics across all performance measures, indicating additional predictive value beyond clinical covariates. AUC, area under the receiver operating characteristic curve; SD, standard deviation; F1, F1-score; MCC, Matthews correlation coefficient

Given the potential mechanistic relevance of metabolomic profiling, we next evaluated whether incorporating plasma and fecal NMR-derived metabolites improved prediction of weight response at the 12-week follow-up. We extended the feature set by including all available metabolomic variables alongside the clinical and microbiome features. Feature importance analysis again identified *Oscillibacter sp.* 57_20 as the strongest predictor, while glutamic acid and glutamine concentrations in both plasma and stool, as well as formic acid, ranked among the highest-scoring metabolite features (Additional file 2: Figure S7). To further assess their predictive contribution, we evaluated metabolite-containing models in multiple combinations. Despite their high importance within the extended model, inclusion of metabolomic variables did not improve predictive performance in repeated cross-validation. The original model combining clinical and microbiome features achieved the highest performance, whereas models including metabolomic features showed consistently lower AUC and accuracy (Additional file 2: Supplementary Table S5). Finally, we examined the abundance patterns of the four features from the reduced model within our cohort. Responders exhibited significantly higher levels of *Oscillibacter sp.* 57_20 (Fig. 3d), while the identified *Faecalibacterium sp.* showed no significant difference (Fig. 3f). Although systolic BP and LDL contributed to model classification, their individual baseline levels did not differ significantly between responders and non-responders (Fig. 3e, g).

Taken together, we identified four (two clinical and two microbiome) features that jointly hold the potential to predict long-term weight loss in response to prolonged fasting intervention. Although performance remained consistently above random across repeated cross-validation, we emphasize the expected variability due to small cohort size and interpret the findings as exploratory but reproducible evidence of predictive potential.

#### Application of the classifier in independent cohorts

To test transferability in independent datasets, we applied the final model to data from three publicly available cohorts that differed in health status and fasting design. Given these differences, these analyses are treated as exploratory applications rather than formal external validations.

**Fig. 4:**
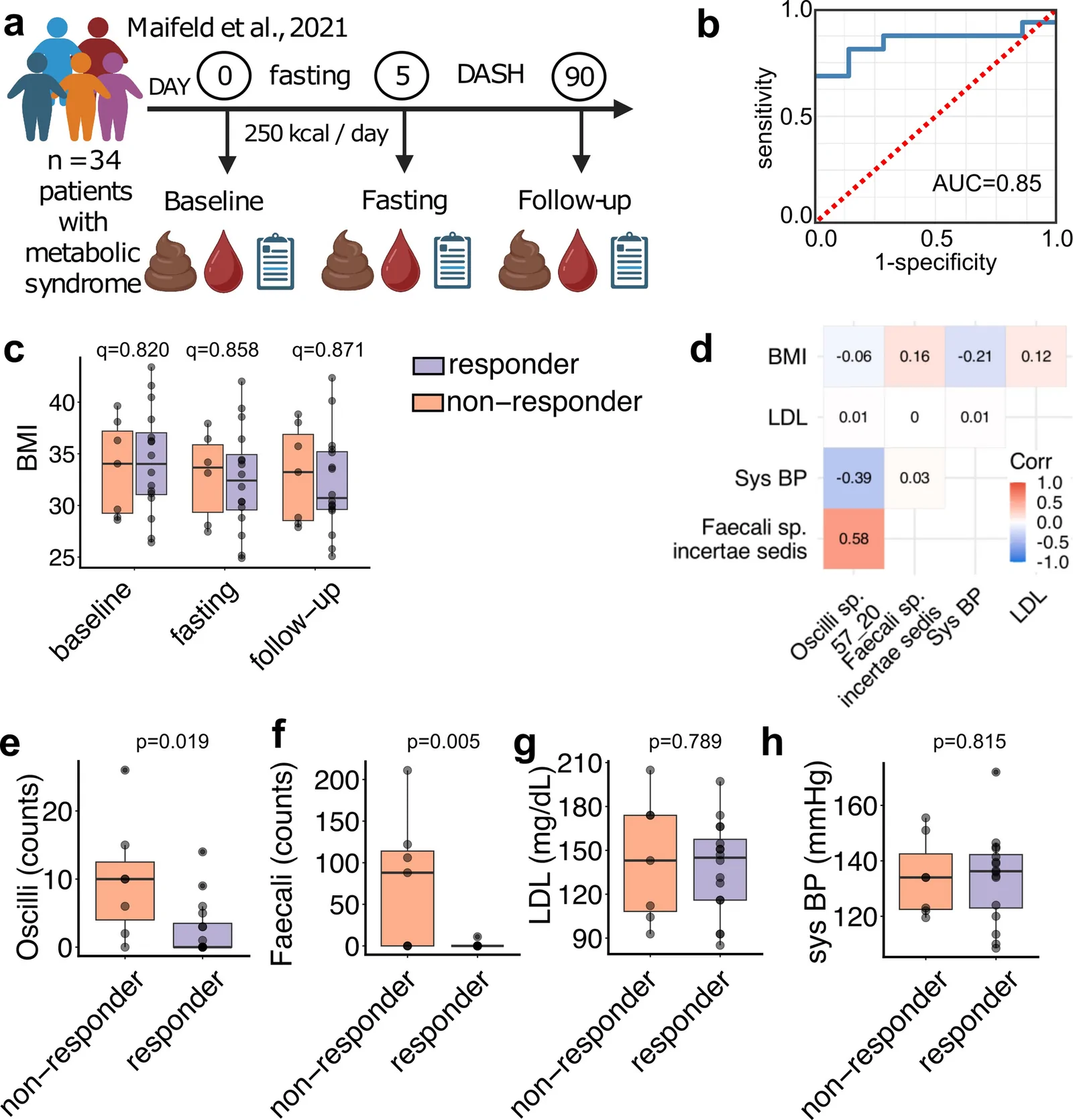
Application of BMI response prediction model in an external fasting cohort of patients with metabolic syndrome. **a**) Schematic of the Maifeld et al. (2021) study: 5-day fasting followed by a DASH diet in individuals with metabolic syndrome (*n* = 34), with BMI follow-up after 12 weeks. **b**) Receiver operating characteristic (ROC) curve of model performance in the external Maifeld cohort, showing good predictive accuracy (AUC = 0.85). **c**) BMI over the course of the study, split by participants classified as responders or non-responders. Each point represents a participant. P-values are from paired Wilcoxon signed-rank tests and were corrected for multiple comparisons using the Benjamini-Hochberg false discovery rate (FDR-BH) method. **d**) Correlation heatmap of model features with BMI. Features used for classification at baseline: **e**) *Oscillibacter sp.* 57_20, **f**) *Faecalibacterium sp.* incertae sedis, **g**) LDL, and **h**) systolic blood pressure. Oscilli, *Oscillibacter sp.* 57_20; Faecali, *Faecalibacterium sp.* incertae sedis; LDL, Low-density lipoprotein cholesterol; sys BP, systolic blood pressure

As a first step, we applied our model to our published metabolic syndrome cohort, in which patients underwent either a five-day fasting regimen followed by a DASH diet or a DASH diet alone (Fig. 4a, *n* = 34) [7]. The study outcomes were assessed 12 weeks post-intervention, mirroring our study’s timeline. Given the greater BMI variability expected in metabolic syndrome patients, we quantified BMI fluctuation from the DASH only group (control group). Responders were again defined as exceeding the mean plus two standard deviation weight loss of the control group (DASH only), corresponding to BMI reduction exceeding 0.8 kg/m^2^. Remarkably, our trained model predicted sustained weight loss in this study with an AUC of 0.85 (Fig. 4b-c, Additional file 2: Supplementary Table S6). Next, we calibrated the model using Platt scaling and isotonic regression. Despite stable strong discrimination, the Brier score remained unchanged after calibration (Additional file 2: Supplementary Table S6), indicating that residual error likely reflects differences in outcome prevalence or underlying data distributions rather than miscalibration alone. Consistent with our healthy-participant cohort, the model’s predictive features exhibited only weak correlations with baseline BMI, underscoring their independence from initial body mass (Fig. 4d). In univariate analyses, *Oscillibacter sp.* 50_27 and *Faecalibacterium sp.* showed significant differences at baseline between responders and non-responders, while clinical features showed no difference (Fig. 4e-h).

**Fig. 5:**
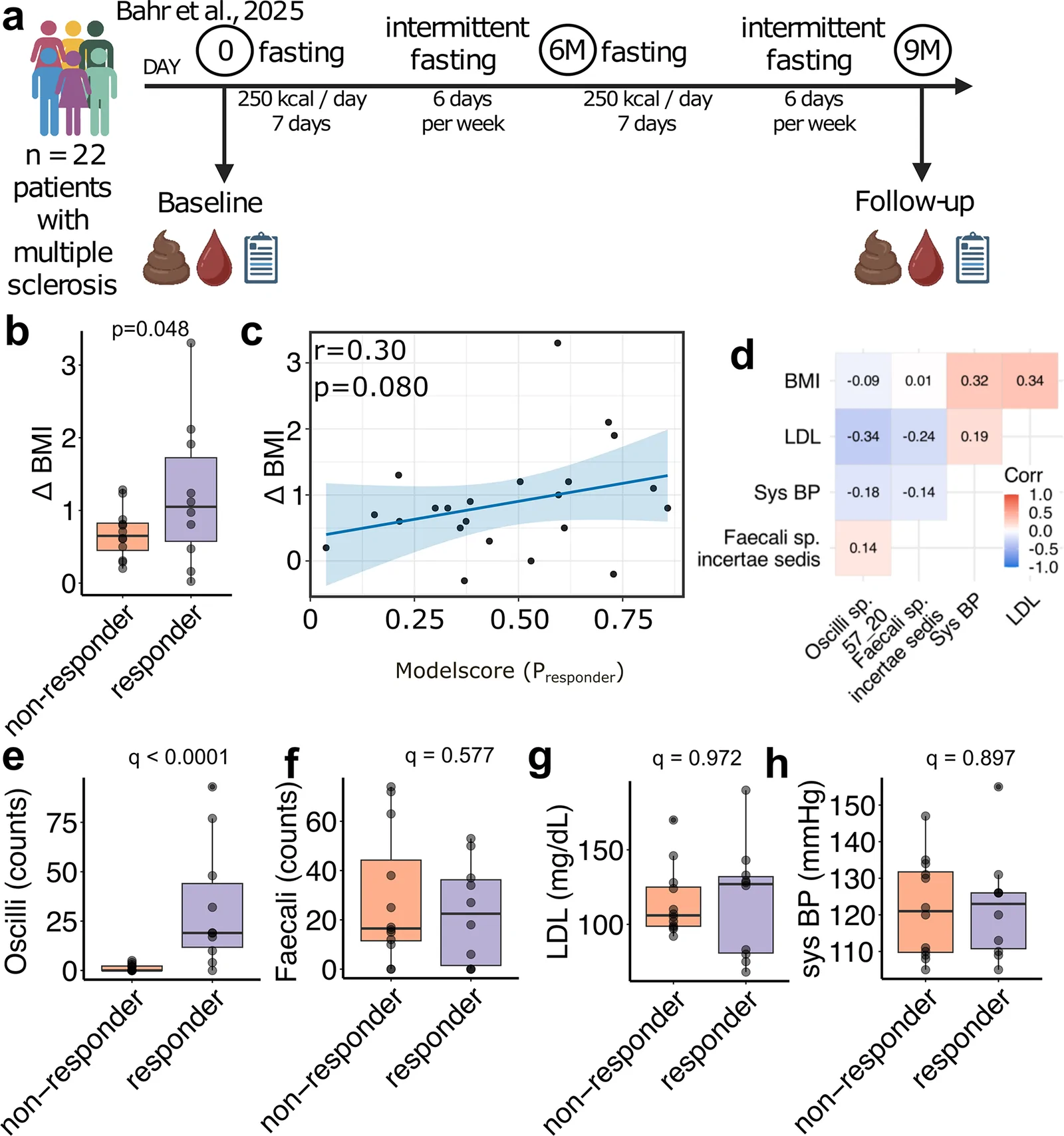
Application of BMI response prediction model in an external fasting cohort of patients with multiple sclerosis. **a**) Schematic of the Bahr et al. (2025) study: two 7-day periods of prolonged fasting and intermittent fasting in individuals with multiple sclerosis (*n* = 22), over a 9-month period. **b**) BMI reduction at follow-up of patients classified as responder or non-responder by the prediction model. p-value by one-tailed unpaired t-test. **c**) Correlation analysis of changes in BMI with probability for responder status derived from the model prediction. P-value by one-tailed Spearman correlation. **d**) Correlation heatmap of model features with BMI. Features used for classification at baseline: **e**) *Oscillibacter sp.* 57_20, **f**) *Faecalibacterium sp.* incertae sedis, **g**) LDL, and **g**) systolic blood pressure. Oscilli, *Oscillibacter sp.* 57_20; Faecali, *Faecalibacterium sp.* incertae sedis; LDL, Low-density lipoprotein cholesterol; sys BP, systolic blood pressure

**Fig. 6:**
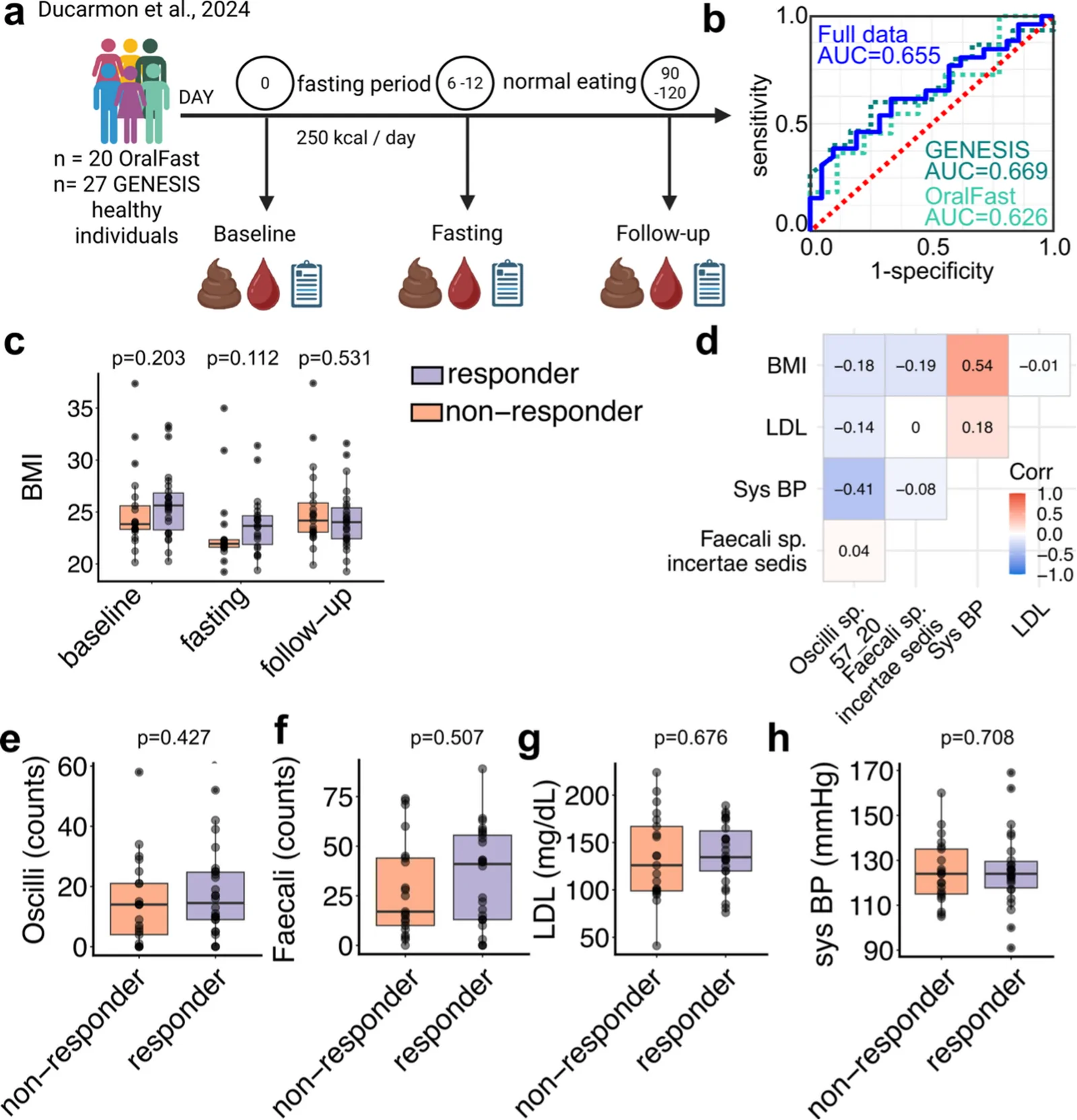
Application of BMI response prediction model in an external fasting cohort of healthy volunteers. (**a**) Schematic of the Ducarmon et al. (2024) study: 6-12-days prolonged fasting with BMI follow-up after 3 months in the OralFast subcohort (*n* = 20) or 4 months in the GENESIS subcohort (*n* = 27). (**b**) Receiver operating characteristic (ROC) curve of model performance showing intermediate accuracy (AUC = 0.655). (**c**) BMI over the course of the study, split by participants classified as responders or non-responders. Each point represents a participant. P-values are from paired Wilcoxon signed-rank tests and were corrected for multiple comparisons using the Benjamini-Hochberg false discovery rate (FDR-BH) method. (**d**) Correlation heatmap of model features with BMI. Features used for classification at baseline: (**e**) *Oscillibacter sp.* 57_20, (**f**) *Faecalibacterium sp.* incertae sedis, (**g**) LDL, and (**h**) systolic blood pressure. Oscilli, *Oscillibacter sp.* 57_20; Faecali, *Faecalibacterium sp.* incertae sedis; LDL, Low-density lipoprotein cholesterol; sys BP, systolic blood pressure

As a further dataset, we applied the classifier to a recent nutritional intervention trial in patients with multiple sclerosis, the majority of whom exhibited normal metabolic profiles (obesity in one patient, hypertension in two, and elevated LDL in four) [26]. Notably, the study design differed from the other cohorts. Patients completed two prolonged 7-day periods of fasting (Fig. 5a, *n* = 22). However, these prolonged fasting periods were interspersed with intermittent fasting (14 h/ day), and follow-up assessments were conducted nine months apart (Fig. 6a). Because no control arm was available to isolate the effects of the prolonged fasting periods from those of intermittent fasting, we could not define average BMI variation and thus compute specificity and sensitivity or perform calibration. However, patients classified as responders by our model experienced significantly greater weight loss than those classified as non-responders (Fig. 5b). To provide an alternative quantitative assessment, we evaluated the correlation between the probability of responder classification and the observed magnitude of BMI change (Fig. 5c). While the Spearman correlation did not achieve statistical significance (*p* = 0.08), the direction and magnitude of the association (ρ = 0.309) support the model’s ability to reflect observed BMI changes. No significant correlations between model parameters and baseline BMI were detected (Fig. 5d). Similar to the current cohort, levels of *Oscillibacter sp.* 50_27 were significantly higher in responders (Fig. 5e), while the other features did not differ significantly (Fig. 5f-h).

Lastly, we applied the model to available data from healthy volunteers undergoing between 6 and 12 days of prolonged fasting [27]. This dataset comprises two subcohorts: the GENESIS (ClinicalTrials.gov: NCT05449249) and OralFast (ClinicalTrials.gov: NCT05031598) trials. Both follow similar study designs (Fig. 6a) but differ in their follow-up durations — 3 months for OralFast and 4 months for GENESIS. Outcome was assessed after 16 weeks during a study visit and as self-reported outcome after 12 weeks, respectively (Fig. 6a, *n* = 47). Since this study was performed in healthy participants, we used the same BMI cut-offs for responder definition as used for the model training in our cohort. Our trained model predicted sustained weight loss in this study with an AUC of 0.655. (Fig. 6b-c, Additional file 2: Supplementary Table S7). Of note, AUC was slightly better for the GENESIS than the OralFast data (Fig. 6b). Platt scaling and isotonic regression did not improve model calibration but retained consistent discrimination (Additional file 2: Supplementary Table S7). In contrast to the other cohorts, baseline BMI and systolic blood pressure were correlated (Fig. 6d). In univariate analyses, none of the features differed significantly at baseline between responders and non-responders (Fig. 6e, h).

Our findings indicate that baseline patient characteristics determine the magnitude of weight response at the 12-week follow-up after prolonged fasting interventions. Furthermore, the predictive model, initially developed in healthy subjects, was successfully applied to independent cohorts investigating prolonged fasting. However, given the differences in health status, study design, and the absence of an intermittent fasting-only group in the multiple sclerosis cohort, this represents an exploratory rather than a formal validation. The observed association between predicted responder status and actual BMI reduction provides supportive but not confirmatory evidence of external applicability, highlighting the need for larger, harmonized fasting studies to confirm generalizability.

#### Metabolite associations with *Oscillibacter sp*. 57_20 and *Faecalibacterium sp*. incertae sedis

**Fig. 7:**
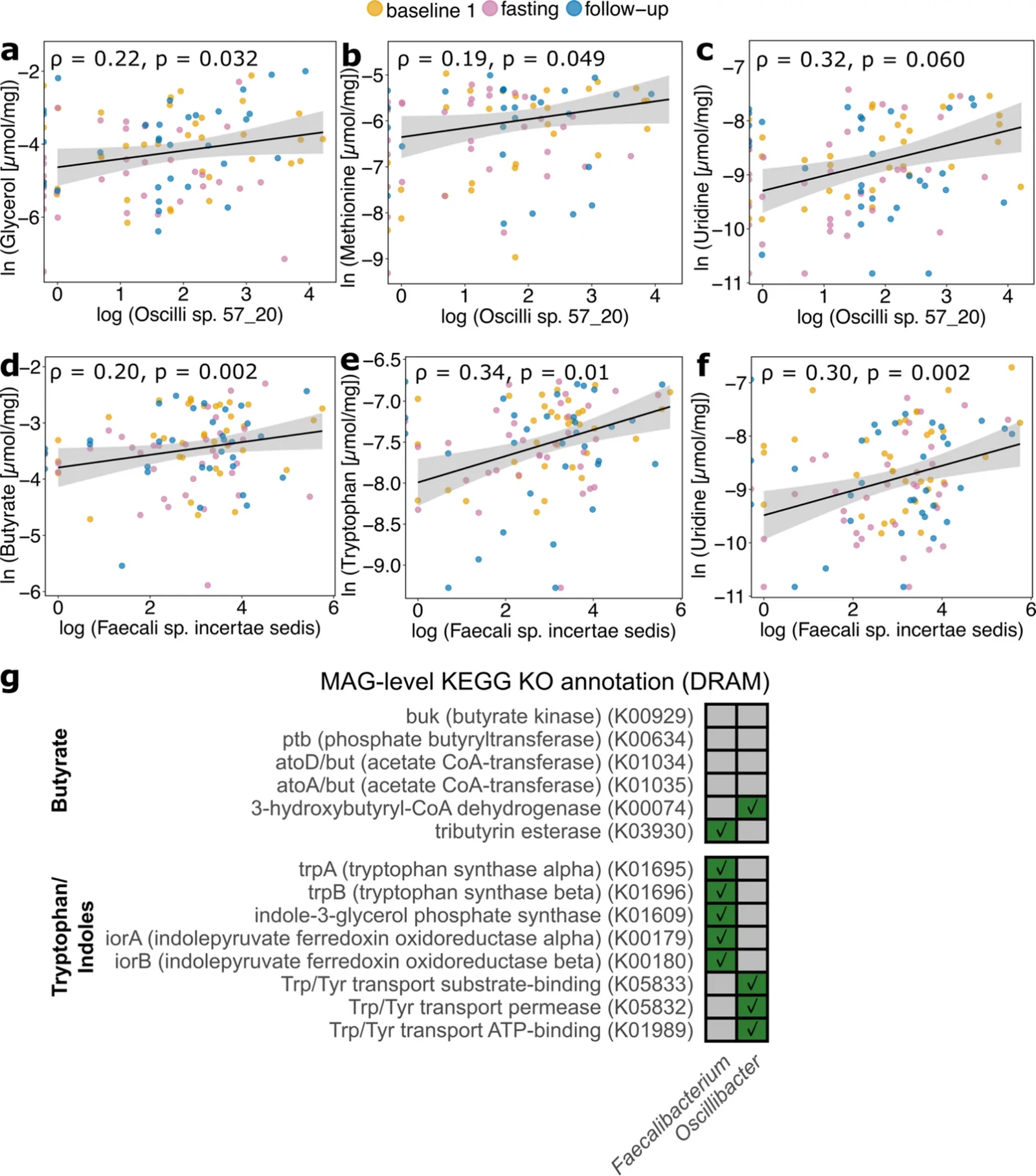
*Oscillibacter sp.* 57_20 and *Faecalibacterium sp.* incertae sedis abundance correlates with fecal metabolome. Correlation of *Oscillibacter sp.* 57_20 abundance with fecal (**a**) glycerol, (**b**) methionine, and (**c**) uridine levels. Correlation of *Faecalibacterium sp.* incertae sedis abundance with fecal (**d**) butyrate, (**e**) tryptophan, and (**f**) uridine levels. R-values from Spearman’s correlation. P-values from permutation-based independence test screening for associations of species with metabolites while blocking effects of study visits. (**g**) Metagenomic assembled genomes (MAG) were analyzed for the presence of genes enoding for tryptophan and butyrate metabolism. Abbreviations: Oscilli: *Oscillibacter sp.* 57_20; Faecali: *Faecalibacterium sp.* incertae sedis

Since both the *Oscillibacter sp.* 57_20 and the *Faecalibacterium sp.* identified in the LEANER were predictive of weight loss, we wanted to further understand the potential metabolite profile associated with these bacteria. Therefore, we performed an exploratory correlation analysis with the fecal metabolome across all visit data while blocking the visit information [31]. We found significant associations of *Faecalibacterium sp.* with butyrate, tryptophan, and uridine (Fig. 7d-f, Additional file 9) as well as of *Oscillibacter sp.* 57_20 with glycerol and methionine, and for uridine without reaching statistical significance (Fig. 7a-c, Additional file 10). To complement this analysis, we performed metagenome-assembled genomes (MAG)-level analysis. Matching the correlation analysis the *Faecalibacerterium sp.* encodes several enzymes for butyrate synthesis and tryptophan catabolism (Fig. 7g). Interestingly, despite the lack of correlation, *Oscillibacter sp.* 57_20 similarly possesses genes encoding for butyrate synthesis but only encodes tryptophan transport proteins (Fig. 7g). In summary, our data suggest that the bacterial species important for classifying responders at the 12-week follow-up modulate the metabolomic output of the fecal microbiome.

## Discussion

Prolonged fasting over several consecutive days has been studied less extensively than continuous energy restriction and intermittent fasting regimens, including time-restricted eating, alternate-day fasting, and whole-day or 5:2 fasting. Randomized studies and meta-analyses generally indicate that intermittent and continuous energy restriction achieve comparable reductions in body weight and improvements in selected cardiometabolic outcomes, although effects vary across protocols and intervention durations [32–35]. In contrast, evidence on prolonged fasting has largely been derived from observational studies or smaller clinical studies, frequently involving participants with metabolic disease [36–38].

The LEANER study adds randomized, waitlist-controlled evidence on a 5-day prolonged fasting intervention in healthy adults. Reductions in BMI and fat mass remained detectable at 12-week follow-up, whereas most cardiometabolic, microbiome, and metabolomic alterations observed directly after fasting were transient and largely reverted after refeeding. This divergence indicates that acute molecular responses to fasting do not necessarily represent mediators of sustained changes in body weight. Direct comparison with continuous energy restriction or intermittent fasting is limited by differences in intervention intensity, duration, and follow-up and, importantly, by the absence of an active dietary comparator in LEANER. The study therefore does not establish superiority of prolonged fasting or distinguish effects attributable specifically to fasting from those of acute energy restriction, refeeding, or subsequent behavioral changes.

Since a strong metabolic phenotype was expected, REE reduction was predefined as primary endpoint. REE in fasting was studied previously mainly in healthy men [39–41]. Our data shows an overall similar but smaller effect, which might be due to the inclusion of healthy women and a broader range of lean masses. REE reduction after 24 h caloric deprivation was previously associated with weight gain after 6 months in healthy individuals [42]. We could not confirm these findings in our cohort with respect to weight changes at the 3-month follow-up using changes in REE after 5 days of fasting (Additional file 2: Figure S8). While blood glucose dropped during fasting, it increased upon refeeding during the day. Since values at night did not increase, our data suggest a mildly disturbed glucose handling for the early days post-fasting. A similar phenotype was reported in the literature in response to standardized meal intakes after prolonged fasting [43, 44]. This was attributed to the metabolism successfully switching to fatty acid and ketone oxidation and therefore reduced postprandial carbohydrate oxidation [43].

Interindividual variability in response to dietary interventions poses a major challenge in lifestyle interventions. Large-scale studies have shown that both host and microbial factors shape individual metabolic responses, emphasizing the need for precision strategies [45, 46]. Based on baseline microbiome and clinical data, we can predict individuals who will respond to fasting with weight loss at 12-week follow-up. Using available data from similar interventions in healthy volunteers, patients with metabolic syndrome, and patients with multiple sclerosis, we could apply our data-driven classification approach. Taken together, our findings indicate that baseline gut microbiome and clinical features contain predictive information on BMI changes following prolonged fasting and that this signal remains detectable across cohorts differing in health status and intervention design. We used a data-driven definition of responders, defining weight loss beyond the mean plus two standard deviations of the control group in each cohort. This represents a statistical rather than a clinical threshold, chosen to capture changes exceeding normal physiological variation [30]. While this approach ensures objectivity across cohorts, it may not fully reflect clinically meaningful improvements, which future studies should define more precisely.

Our prediction results extend a growing literature indicating that baseline gut microbiome features may stratify weight-loss responses across dietary interventions. In overweight adults, a higher pretreatment Prevotella-to-Bacteroides ratio was associated with greater weight and fat loss during a 24-week energy-restricted diet, particularly at higher dietary fiber intake [47]. Grembi et al. further reported that microbiome plasticity, rather than a reproducible baseline taxonomic signature, was associated with sustained 12-month weight loss during low-fat or low-carbohydrate diets [48]. At the functional level, Diener et al. identified baseline metagenomic features related to bacterial replication, substrate degradation, stress responses, respiration, and cell-wall synthesis that were associated with subsequent weight change in a selected subgroup of a lifestyle-intervention cohort [49]. Similarly, Dong et al. used baseline microbiome profiles to classify participants who achieved at least 5% weight loss during calorie restriction, although the model was evaluated in a small cohort without external validation [50]. In this context, our findings extend microbiome-based response prediction to prolonged fasting and indicate that baseline microbial and clinical features contain information on the 12-week BMI response following a 5-day fasting intervention. The performance of the classifier in independent fasting cohorts provides preliminary support for the external generalizability of the predictive signature across populations differing in health status. However, predictive importance does not establish mechanistic involvement.

We have shown in the past the microbiome and immune data can predict blood pressure reduction in patients with metabolic syndrome in response to fasting [7]. In contrast, our study in healthy participants revealed only modest effects on blood pressure at 12-week follow-up. Given the well-established impact of fasting on weight management [51], we applied a data-driven approach based on BMI changes. Our initial model, which incorporated 83 features, demonstrated high sensitivity but limited specificity. Iterative feature selection enabled us to identify *Faecalibacterium sp.* incertae sedis, *Oscillibacter sp*. 57_20, plasma LDL levels, and systolic blood pressure for a feature-reduced classifier. When extending the data to NMR metabolomics, glutamine, glutamic acid, and formic acid ranked among the most important metabolite features in the extended model. However, adding plasma and fecal metabolomics did not improve out-of-sample performance beyond the combined clinical and microbiome model. This potentially indicates that the metabolite profiles captured biological variation already represented by the clinical and microbial features. Nevertheless, their consistent ranking suggests that amino acid and microbial metabolic pathways may be associated with the 12-week response to prolonged fasting and warrant further investigation in larger cohorts with matched metabolomic data.

Application of the classifier to independent cohorts provided heterogeneous but supportive evidence of transferability. Discrimination was strongest in the metabolically affected cohort with a closely matched intervention and follow-up design [7], whereas performance was attenuated in cohorts [26] differing in fasting duration, follow-up timing, outcome assessment, and participant characteristics. In the multiple-sclerosis cohort, the absence of an intermittent fasting-only control precluded formal evaluation of discrimination and calibration. In the Ducarmon dataset [27], with healthy participants and substantially longer fasting periods, performance was moderate and differed between the GENESIS and OralFast subcohorts, which varied in follow-up duration and outcome assessment, including self-reported weight in OralFast. These applications should therefore be considered exploratory rather than formal external validations and indicate that model transportability depends on harmonized interventions, measurements, and outcome definitions.

To our knowledge, no prior study has directly linked *Oscillibacter sp.* 57_20 with weight regulation. However, *Oscillibacter sp.* 57_20 was recently identified as a keystone taxon in a similar prolonged fasting study in healthy individuals, where it was implicated in the production of indole-3-propionate (I3P) [27], a protective metabolite in cardiometabolic disease [52]. Future research is needed to explore the mechanistic role of *Oscillibacter spp.* in host metabolism and its potential relevance in personalized fasting interventions. *Faecalibacterium spp.* are known SCFA producers [53]. In line, our data shows that the abundance of our specific *Faecalibacterium sp.* associates with fecal butyrate levels. As described previously for other *Faecalibacterium spp.* [54], the identified *Faecalibacterium sp.* correlates with fecal tryptophan. Tryptophan can be catabolized by gut microbiota to indole metabolites [55]. Unfortunately, due to the resolution of NMR metabolomics, we could not detect specific indole metabolites like I3P.

Both the *Faecalibacterium sp.* identified in this study and the *Oscillibacter sp.* 57_20 correlate with the nucleoside uridine. Uridine can adaptively regulate food intake in healthy participants [56] and reflects the nutritional status of mice [57, 58] with high uridine levels as indicators of catabolic states [57, 59]. In line, dietary supplementation of uridine enhanced hunger and increased caloric intake [56]. The observed associations with uridine may warrant further investigation in relation to nutritional state and appetite regulation. Unfortunately, the literature concerning the other metabolites of interest correlating with *Oscillibacter sp.* 57_20, glycerol and methionine is sparse. Because these analyses were exploratory and performed while conditioning on study visit, the resulting associations should be interpreted as hypothesis-generating rather than mechanistic evidence. Although this conservative approach reduces statistical power in a cohort of this size, it minimizes confounding by fasting-induced temporal changes and provides a more robust basis for future mechanistic investigations. Of note, MAG analysis indicated that the identified *Faecalibacterium sp.* encoded enzymes involved in butyrate synthesis and tryptophan to I3P catabolism, whereas the corresponding enzymes were not identified in *Oscilibacter sp.* 57_20.

Overall, the gut microbiome and metabolome exhibited a consistent pattern of fasting-induced alterations and nearly complete reversion to baseline at follow-up. Previous studies have reported enrichment of SCFA-prodcung bacteria, including as *F. prausnitzii* [60–62], as well as *Roseburia* and *Coprococcus* species [63–65]. Our findings only partly overlapped with these reports, as we observed no alterations to *F. prausnitzii*, alongside a decrease in *Roseburia sp.* and *Coprococcus sp.* However, one *Butyricicoccus sp.* increased and functional analysis indicated enhanced propionate production via kinase pathways, suggesting that fasting-induced changes in SCFA production may reflect coordinated community-level effects rather than changes in individual taxa. Differences from previous studies may partly relate to the healthy status and baseline microbiome composition of our cohort, suggesting that fasting may have stronger effects in initially disturbed microbial communities. Interestingly and consistent with previous studies, we did not observe an overall change in alpha diversity [7, 64]. However, participants with lower baseline alpha diversity showed a sustained increase after fasting intervention. Because higher microbial diversity may stabilize microbiome functions during perturbations [66] and fasting interventions have increased alpha diversity in such as obesity [67], these studies reinforce that diversity responses are more pronounced in individuals with lower baseline diversity. Nevertheless, low baseline alpha diversity may also arise from unaccounted external influences, including lifestyle factors and medication.

Despite these promising results, our study has several limitations. First, the lack of comprehensive documentation on dietary intake following the fasting intervention. Whether significant weight loss was solely due to fasting or also influenced by subsequent dietary modifications, a common limitation in caloric restriction studies cannot be determined. From a computational perspective, the relatively small sample size, particularly the initial test dataset after splitting, poses challenges for the random forest model and feature selection [68]. However, the testing of the classifier in three independent cohorts shows the robustness of our approach [69].

## Conclusions

Our study provides compelling evidence that fasting induces substantial yet reversible changes in the gut microbiome and host metabolic profiles. Importantly, a subset of individuals experiences sustained weight loss. The integration of microbial and clinical data not only enhanced our predictive capabilities but also unveiled novel associations, such as the independent role of *Oscillibacter sp.* 57_20 and *Faecalibacterium sp.* incertae sedis in weight regulation. Despite limitations related to dietary documentation and sample size, these findings pave the way for future research aimed at refining personalized fasting interventions. Ultimately, our work underscores the promise of leveraging gut microbiome insights to tailor dietary strategies for improved metabolic health and weight management.

## Supplementary Information

Below is the link to the electronic supplementary material.

Supplementary Material 1: Additional file 1: English translation of the approved study protocol.

Supplementary Material 2: Additional file 2: Supplementary Tables S1-S7. Supplementary Figures S1-8.

Supplementary Material 3: Additional file 3: Statistical comparisons and descriptive statistics of clinical data.

Supplementary Material 4: Additional file 4: Statistical comparisons and descriptive statistics of bacterial species.

Supplementary Material 5: Additional file 5: Statistical comparisons and descriptive statistics of gut-specific metabolic modules.

Supplementary Material 6: Additional file 6: Statistical comparisons and descriptive statistics of plasma metabolites.

Supplementary Material 7: Additional file 7: Statistical comparisons and descriptive statistics of fecal metabolites.

Supplementary Material 8: Additional file 8: Feature list used for machine learning.

Supplementary Material 9: Additional file 9: Correlation analysis of *Faecalibacterium sp*. incerta sedis with fecal metabolites.

Supplementary Material 10: Additional file 10: Correlation analysis of Oscillibacter sp. 57_20 with fecal metabolites.

## Data Availability

Analysis code, scripts, and processed raw data can be accessed on Zenodo (https://doi.org/10.5281/zenodo.17493808) [70]. Raw sequencing data is available under the BioProject ID PRJNA1293883 from the NIH Sequence Read Archive (https://www.ncbi.nlm.nih.gov/bioproject/PRJNA1293883) [71].
