# Supplementary material for: Machine learning identifies microbiome and clinical predictors of sustained weight loss following prolonged fasting
Source: Genome Med. 2026 Sep 16;18:136. doi: 10.1186/s13073-026-01765-0 (PMC13587497; doi:10.1186/s13073-026-01765-0)
Supplement: Supplementary file 1 [file 13073_2026_1765_MOESM1_ESM.docx]

**Concise English translation of the study protocol approved by the Charité Ethics Committee (LEANER Study; approval granted on 26 May 2020)**

| 1. Title of the Study | Physiological Changes Induced by Five Days of Fasting in Healthy Adults (LEANER). |
| --- | --- |
| 2. Ethics Committee Application Number | EA1/033/20 |
| 3. Decisions by Other Ethics Committees | No application has been submitted to any other ethics committee. |
| 4. Study Objective and Hypotheses | The aim of this project is to investigate the effects of a 5-day Buchinger fasting intervention on energy metabolism, body composition, glucose variability, blood pressure, gut microbiome composition, immune system parameters, emotional processing, and food-related reward delay in healthy participants. In addition, we aim to examine whether potential fasting-induced effects persist three months after the intervention. The following hypotheses will be tested.  **Primary hypothesis**  Five days of fasting reduces resting energy expenditure in the fasting state compared with baseline.  **Secondary hypotheses**  Five days of fasting alter substrate oxidation, body weight, body composition, blood pressure, the composition and metabolic activity of the gut microbiome, the frequency of pro-inflammatory immune cells, glucose variability following standardized meals, as well as neurocognitive correlates of emotional processing and food-related reward delay. Some of these effects are expected to persist up to 12 weeks after completion of the fasting intervention.  **Primary endpoint**   - Resting energy expenditure (kcal/day), measured by indirect calorimetry.   **Secondary endpoints**   - Respiratory exchange ratio in the fasting state, measured by indirect calorimetry - Office blood pressure (mmHg) - Frequency of bacterial species in stool samples - Frequency of bacterial metabolites in stool samples - Frequency of bacterial and human metabolites in blood - Frequencies of peripheral immune cell populations (%) - Interstitial glucose concentration (mg/dl) - Neurocognitive correlates of emotional processing (processing of general affective stimuli) - Neurocognitive correlates of food-related reward delay, both assessed using functional MRI - Perceived stress, measured using the Perceived Stress Questionnaire (PSQ) - Self-efficacy, measured using the General Self-Efficacy Scale (SWE) - Physical activity, assessed by questionnaire - Safety and tolerability of fasting   All endpoints will also be analyzed stratified by sex. |
| 5. Scientific Background and Significance | Fasting is defined as the body's ability to meet its nutritional needs for a limited period of time from its own energy reserves without endangering health. Fasting refers to a voluntary and temporary abstinence from solid food and stimulants such as alcohol, caffeine, and nicotine. Interest in fasting is rapidly increasing in times of overnutrition, overweight, and a high prevalence of diet-related diseases.  The German physician Otto Buchinger was among the first to systematically observe and document the effects of fasting on a wide range of diseases and developed the concept of therapeutic fasting. In the present study, we intend to have healthy volunteers fast for five days according to the method developed by Buchinger.  For Buchinger fasting, there is already level 1A evidence supporting its use in the treatment of Rheumatoid Arthritis. Preliminary evidence from smaller non-randomized or observational studies exists for conditions such as Metabolic Syndrome, Osteoarthritis, Fibromyalgia, Hypertension, chronic musculoskeletal pain, Migraine, and as a supportive measure during Cancer chemotherapy^1^. However, to date there have been very few studies investigating this type of fasting in healthy individuals.  The aim of our study is to characterize both clinical endpoints and mechanisms of fasting. In addition to obvious endpoints such as energy metabolism, body weight, body composition, and blood pressure, we also aim to investigate more specific outcomes, including the composition of the gut microbiome, metabolomics in blood and stool, and the effects on the immune system.  Two studies have already investigated the microbiome in healthy individuals undergoing fasting. One study showed that a one-week fasting program followed by a six-week probiotic intervention increased microbial diversity in overweight participants^2^. However, due to this design, the independent effects of fasting and their persistence remained unclear.  A more recent study from the Buchinger Wilhelmi Clinic analyzed the effects of ten days of fasting on the gut microbiome of 15 healthy men^3^. Limitations of this study included the small sample size, exclusion of women, absence of a control group, and the assumption of participants’ health status based solely on self-report.  The metabolome of healthy participants after 12 and 36 hours of fasting has already been investigated^4^. However, after 36 hours the body has not yet fully transitioned into the fasting metabolic state. In contrast, we aim to analyze the metabolome after the body has fully switched to fasting metabolism.  None of these studies investigated the interaction between fasting, the gut microbiome, and the immune system, nor the role of bacterial metabolites that act as messengers between these two systems.  Another distinctive feature of our study is the continuous glucose monitoring over 14 days, which will allow the glucose metabolism before, during, and after fasting to be characterized in detail for the first time. This will enable an assessment of gluconeogenesis activity. At the same time, participants will measure their ketone body concentrations daily during fasting.  Reports from individuals who practice fasting suggest that fasting may improve mood and eating behavior for weeks to months after the fasting period^5,6,7^. However, no studies have yet examined the neurocognitive basis of such changes using the Buchinger fasting method. Against this background, we aim to investigate longitudinal neurocognitive changes in emotional processing and reward delay related to eating behavior using functional MRI.  Overall, our objective is to generate a comprehensive understanding of the physiological changes induced by fasting in healthy humans. This knowledge is relevant both for individuals who already fast regularly to prevent chronic diseases and for improving our understanding of physiological processes that may form the basis for the treatment of chronic diseases. |
| 6. Applicable Legal Regulations | EU General Data Protection Regulation (GDPR) and Berlin Data Protection Act (BlnDSG). |
| 7. Description of the Intervention | Buchinger fasting will be supervised by trained fasting instructors in groups of 5–10 participants. During the five fasting days, participants consume approximately 200–250 kcal per day through vegetable juices and vegetable broths. Tea and water may be consumed ad libitum.  Two preparatory days precede the fasting period and three refeeding days follow the fasting period. Participants are encouraged to take time off work during the fasting week. Bowel cleansing procedures, physical activity, and relaxation exercises are part of the fasting program. |
| 8. Preclinical Data | Not applicable. |
| 9. Previous Studies | See background section (5) above. |
| 10. Study Design and Methods | **Study Design**  This study is designed as a randomized parallel-group trial with a waitlist control group. Participants randomized to the fasting arm will undergo three study visits: before the start of fasting (V1), after five days of fasting (V2), and 12 weeks after fasting (V3). The control group will initially undergo two visits: at baseline (V1) and after 12 weeks (V2). Four weeks later, these participants will cross over to the fasting arm and will then undergo three additional visits (V3–V5) (Fig.1).  **Screening**  Prior to study inclusion, a screening examination will be performed to document the health status of participants. The screening includes medical history, physical examination, body weight and height measurements, hip and waist circumference, electrocardiography (ECG), blood pressure and heart rate measurements, as well as routine laboratory parameters from blood and urine.  **Medical history**  Medical history will be collected using standardized forms. The study physician will review all relevant information with the participants and complete the documentation as necessary.  **Physical examination**  The physical examination includes inspection, percussion, and palpation of the head, neck, thorax, abdomen, spine, and extremities.  **Anthropometric parameters**  Anthropometric parameters will be assessed in a standardized manner.  *Body weight*  Body weight will be measured on the same medically calibrated and TÜV-certified scale at the same time of day with participants wearing underwear.  *Body height*  Body height will be measured at the first visit in the morning without shoes using a medically calibrated stadiometer.  *Body mass index (BMI)*  The body mass index (BMI) will be calculated as body weight in kilograms divided by height in meters squared.  *Waist circumference*  Waist circumference will be measured in the standing position at the midpoint of the abdomen between the lower rib margin and the anterior superior iliac spine.  *Hip circumference*  Hip circumference will be measured in the standing position at the level of the greater trochanter of the femur. All measurements will be performed on lightly clothed participants using a two-meter measuring tape.  *Waist-to-hip ratio*  The waist-to-hip ratio will be calculated as the quotient of waist circumference divided by hip circumference.  **Blood samples**  Blood samples will be obtained for both routine laboratory testing and specific analyses.  *Routine parameters*  Routine parameters, including blood count, glucose, thyroid-stimulating hormone (TSH), blood lipids, electrolytes, aminotransferases, creatinine, coagulation parameters, and urea, will be measured to screen for underlying diseases.  *Specific analyses*  Additional blood samples will be used to investigate immunoregulatory functions of immune cells as well as circulating hormones, cytokines, and metabolites involved in glucose and ketone body metabolism.  **Study protocol**  At each study visit (V1–V5), several assessments will be performed:   - body composition measurement - bioelectrical impedance analysis (BIA) - blood pressure measurement - blood sampling - isolation of peripheral blood mononuclear cells (PBMCs) - indirect calorimetry - completion of questionnaires - magnetic resonance imaging (MRI). The MRI examination will include two functional MRI (fMRI) paradigms assessing emotional processing and reward delay, as well as the acquisition of structural anatomical sequences.   In the days following each study visit (V1-5), additional procedures will be conducted:   - continuous glucose monitoring for seven days - dietary assessment (4 days) - standardized breakfast meals (3 days) - collection of a stool sample (1 day)   **Body composition**  Body composition will be assessed using air-displacement plethysmography (ADP) with the BodPod system (Life Measurement Inc., Concord, CA, USA). In this method, the participant’s body volume is determined through air displacement in a measurement chamber. Combined with the previously measured body weight, body density can be calculated. Using established mathematical model equations, the ratio of fat mass to fat-free mass can subsequently be determined.  **Bioelectrical impedance analysis (BIA)**  Bioelectrical impedance analysis will be performed as an additional non-invasive method to estimate body composition. Measurements will be conducted at a frequency of 50 kHz on all four extremities. The method measures inductive resistance (R) and capacitive resistance (reactance, Xc) in ohms, as well as the phase angle (α) between alternating current and voltage. These measurements allow the calculation of fat-free mass, fat mass, body cell mass, total body water, extracellular water, and intracellular water.  **Blood pressure / heart rate**  Blood pressure and heart rate will be measured non-invasively. In addition to systolic and diastolic blood pressure as well as mean arterial pressure, vascular stiffness parameters such as pulse wave velocity and peripheral resistance will be assessed. Measurements will be obtained five times at two-minute intervals at the study center using an automatic oscillometric device (Mobil-o-Graph, I.E.M. GmbH, Stolberg, Germany) while study personnel are absent.  **Immunoregulatory functions**  To investigate immunoregulatory functions, 50 ml of EDTA blood will be collected for immunophenotyping. Peripheral blood mononuclear cells will be isolated and subsequently analyzed using antibody-mediated staining and flow cytometry. The expression of cytokines, including interleukin-17, interleukin-10, interleukin-22, interferon-gamma, and tumor necrosis factor-alpha, will be assessed in leukocyte populations.  **Metabolomics**  Metabolomic analyses will focus on metabolites of glucose and ketone body metabolism, which will be measured using mass spectrometry. For these analyses, 5 ml of serum will be required.  **Indirect calorimetry**  Indirect calorimetry will be performed to assess energy expenditure as well as the oxidation rates of carbohydrates and fats. During the procedure, the participant rests quietly in a supine position with a ventilated hood placed over the head. The hood is equipped with an inlet and an outlet valve. Fresh air is supplied continuously through the inlet valve, while expired air is collected through the outlet valve. Gas samples from the surrounding room air are analyzed, and the differences between inhaled and exhaled gas concentrations allow calculation of oxygen consumption (VO₂) and carbon dioxide production (VCO₂) in ml/min. These values enable calculation of energy expenditure and substrate oxidation rates. The measurement will be conducted for 40 minutes following a 30-minute resting period.  **Questionnaires**  Four domains of daily life that may potentially be influenced by fasting will be assessed using questionnaires. Emotional well-being will be evaluated using the Positive and Negative Affect Schedule (PANAS), which consists of 20 adjectives describing positive or negative affect that are rated on a five-point scale. The PANAS is intended to assess short-term affective states associated with fasting. Current stress perception will be measured using the Perceived Stress Questionnaire (PSQ), which evaluates subjective stress across 20 items covering the subscales worries, tension, joy, and demands, each rated on a four-point scale. The General Self-Efficacy Scale (SWE) will be used to assess perceived self-efficacy, defined as the belief in one’s own competence and control over life situations. Increased self-efficacy after fasting could indicate positive lifestyle changes. This questionnaire contains ten items rated on a four-point scale. In addition, physical activity during work, household tasks, and leisure time over the previous month will be assessed by questionnaire. All questionnaires will be completed on an iPad at the study center, and the data will be stored in a REDCap database. Completion of the questionnaires requires approximately 15 minutes.  **MRI examinations**  MRI examinations will be conducted between 13:00 and 16:00 at the Berlin Center for Advanced Neuroimaging (BCAN). The last food intake before scanning will be standardized.  fMRI experiments  *fMRI experiment !: Neurocognitive mechanisms of processing general affective stimuli*  The first fMRI experiment investigates neurocognitive mechanisms of processing general affective stimuli and is based on a paradigm described by Erk et al. (2010). In this paradigm, 45 images derived from the International Affective Picture System are presented, including 30 emotionally negative and 15 neutral images matched for content, brightness, contrast, color, and frequency of occurrence. Participants are instructed either to allow the emotional response to occur naturally (passive emotion processing) or to regulate their response by adopting the perspective of a neutral observer (active emotion regulation). Each trial begins with a fixation screen presented for approximately 20 seconds, followed by a two-second instruction cue (“allow” or “regulate”), and then an eight-second presentation of the image. Trials are presented in pseudorandom order. The total duration of the experiment is 22 minutes and 30 seconds. This experiment is followed by a ten-minute break outside the scanner.  *fMRI experiment I!:* *Neurocognitive mechanisms of food-related reward delay*  The second fMRI experiment examines neurocognitive mechanisms of food-related reward delay and is based on paradigms previously developed and published by Weygandt and colleagues (2013, 2015, 2019). During scanning, participants repeatedly choose between a smaller food portion available immediately and a larger portion of the same meal available after a delay. Before scanning, participants select their preferred meal from seven possible options to personalize the paradigm. The meals include typical breakfast and lunch dishes such as tomatoes with mozzarella, fried eggs with tomatoes, muesli with dried fruits, pasta salad, muffins, pizza, and schnitzel. Participants are informed that the decisions are hypothetical and that no food will actually be provided during or after the experiment. The experiment consists of 40 decision sequences. Each sequence begins with a fixation screen presented for four to eight seconds. Subsequently, a small portion labeled “immediately” appears on one side of the screen, while a larger portion of the same meal with a delay indicated in minutes appears on the opposite side. Participants indicate their preferred option by pressing a button. During the feedback phase, an arrow below the selected meal changes color from yellow to red to confirm the choice. The selection and feedback phase lasts six seconds. The experiment lasts eight minutes and is performed twice per study day, resulting in a total duration of 16 minutes. In both fMRI experiments, brain activity will be measured using a T2*-weighted multiband echo planar imaging (EPI) blood-oxygen-level-dependent (BOLD) sequence.  Structural anatomical MRI sequence  Following the functional imaging sessions, a structural anatomical MRI sequence will be acquired. Specifically, a T1-weighted three-dimensional magnetization-prepared rapid gradient echo (MPRAGE) sequence will be obtained to enable preprocessing and analysis of the functional MRI data. This sequence allows determination of brain volumetric parameters and optimization of spatial normalization of functional brain images. The acquisition time is approximately five minutes.  **Continuous glucose monitoring**  Continuous glucose monitoring will be performed for seven days using a sensor (FreeStyle Libre Pro, Abbott). The sensor, originally developed for patients with diabetes to avoid frequent finger-prick glucose measurements, is inserted into the subcutaneous tissue of the dorsal upper arm. In this study, participants will not be able to view their glucose values. After seven days, the sensor will be removed by the participant and returned to the study center.  **Dietary intake assessment**  Dietary intake will be assessed alongside glucose monitoring using a four-day free-record dietary protocol. Based on these records, daily energy intake and nutrient composition will be calculated.  **Stool samples**  Participants will also collect a stool sample at each time point. Samples can be collected at home and should correspond to the first spontaneous bowel movement after fasting or after the study visit. After transferring the sample into two collection tubes, participants will store the tubes at −20°C. Once the study center has been notified, sample collection will be arranged. Bacterial DNA will be isolated from the stool samples for sequencing, and metabolic products will be analyzed using mass spectrometry.  **Ketone body concentration**  During the fasting week, participants will measure their ketone body concentration once daily using a handheld device. A small drop of blood will be obtained by puncturing the side of a fingertip (excluding the index finger) using an automated lancet device. After professional instruction, participants will be able to perform this procedure independently, similar to standard glucose measurements performed by patients with diabetes. The values will be stored in the device and can be retrieved upon return to the study center, thereby providing an objective measure of compliance. |
| 11. Risk‑Benefit Assessment |  |
| a. potential medical benefit to study participants (individual benefit to each patient) | Due to the waitlist design, all participants will benefit from a fasting week guided by specialists. In addition, they will receive a comprehensive health check and close monitoring throughout their participation in the study. Fasting in a group setting has a motivating effect and may encourage participants to undertake fasting again at a later time. Fasting is frequently associated with sustained improvements in lifestyle, particularly with regard to reduced consumption of stimulants and other harmful substances, thereby positively influencing long-term health. |
| b. potential medical benefits for people who may develop the condition in the future (group benefit) | The results of this study would, for the first time, comprehensively characterize the so-called “fasting week for healthy individuals” across multiple relevant domains. In the case of positive outcomes, this would provide the first evidence-based demonstration of preventive effects of fasting in healthy individuals, which could form the basis for future fasting recommendations. |
| c. **Risks** and burdens for study participants (list all in detail) | **5-day fasting**  With regard to the five-day fasting period, a recent study from the Buchinger Wilhelmi Clinic in Überlingen reported on the safety and tolerability of fasting durations between 4 and 21 days in 1,422 normal-weight and overweight individuals. Fasting was associated with a significant improvement in physical and emotional well-being, and 93% of participants reported not experiencing hunger. Mild symptoms such as muscle pain, sleep disturbances, headaches, and hunger occurred primarily during the first days of fasting. In addition, dizziness, daytime fatigue, and increased sensitivity to cold may occur. These side effects are considered normal accompanying phenomena of fasting and can generally be managed well under professional supervision. During the fasting week, participants will be able to contact their fasting supervisor at any time via email or telephone in order to receive advice on managing such symptoms.  **Continuous glucose monitoring**  Continuous glucose monitoring carries a small risk of bleeding at the site of sensor insertion. In the event of persistent bleeding, the sensor must be removed. These sensors are routinely applied and removed independently by patients with diabetes and are generally not noticeable while being worn.  **Blood sampling**  Blood sampling carries a small risk of thrombophlebitis and unsuccessful venipuncture. Thrombophlebitis, if it occurs, can be treated with anti-inflammatory medication. In the event of an unsuccessful venipuncture, the affected vessel will be manually compressed followed by appropriate monitoring. In very rare cases, nerve injury may also occur.  **Magnetic resonance imaging (MRI)**  MRI is a non-invasive procedure widely used worldwide. In the present study, no contrast agents will be administered, and when the relevant exclusion criteria are respected, the procedure does not pose a risk to study participants. Mild side effects such as headaches or discomfort due to the loud noise produced by the scanner may rarely occur. Based on our experience with MRI examinations in healthy individuals, patients with multiple sclerosis, and patients with psychiatric disorders, the scanning duration required for this project (approximately 55 minutes), which will be interrupted by a 10-minute rest period outside the scanner, does not represent an excessive burden for study participants.  No risks are associated with the remaining study procedures. |
| 12. Risk Management | Screening and examinations will be conducted in the Clinical Research Unit of the ECRC with immediate access to emergency care. |
| 13. Withdrawal Criteria | The study may be terminated at any time: • upon withdrawal of consent by the participant • by the study physician for medical or administrative reasons. |
| 14. Number and Demographics of Participants | 40 healthy men and women (1:1 ratio) will be enrolled. |
| 15. Statistical Analysis | The statistical analysis will include only those participants who complete the study in accordance with the study protocol.  **Sample size considerations**  Based on the current state of knowledge in this field, a formal statistical sample size calculation is not feasible. The study therefore has an exploratory character. A sample size of 40 participants in the pre- versus post-fasting comparison and 20 participants in the control versus fasting arm is considered sufficient, based on our experience, to detect relevant effects on the outcome variables under investigation. In addition, the recruitment and examination of 40 participants can be realistically achieved within a reasonable time frame.  **Statistical analysis**  For all outcome variables, the findings will first be explored and analyzed descriptively. Statistical measures such as mean and variance will be calculated for metric variables, median and interquartile range for ordinal variables, and frequencies or proportions for nominal variables.  Normality of the data distribution will be assessed using the Shapiro–Wilk test. Depending on the distribution and study design, data will subsequently be analyzed using repeated-measures analysis of variance (ANOVA) or the Friedman test. Pearson correlation analysis will be used to assess associations between variables.  A p-value < 0.05 will be considered statistically significant. |
| 16.  a. Inclusion and Exclusion Criteria | **Inclusion criteria**   - Men and women in a 1:1 ratio - Age 20–50 years - Body mass index (BMI) 20–30 kg/m² - Existing health insurance that covers the investigation and treatment of any incidental findings   **Exclusion criteria**   - Diseases or functional disorders that, in the opinion of the study physician, preclude participation in the study - Severe, manifest, and treatment-requiring diseases - Continuous use of prescription medications (except hormonal contraception) - Postoperative conditions - Acute or chronic infections - Use of antibiotics within the past six months - Participation in a fasting week within the past six months - Special medical diets - Intake of dietary supplements - Food intolerances - Vegan diet - Pregnancy or breastfeeding - Smoking - Participation in a diet or weight reduction program - Change in body weight of more than 2 kg during the month prior to study entry - Known abuse of medication, drugs, or alcohol - Legal incapacity or other circumstances that prevent the participant from fully understanding the nature, significance, and implications of the study   **Additional exclusion criteria for MRI**  With regard to magnetic resonance imaging, individuals will be excluded if they present with contraindications to MRI, including for example:   - Sensorineural hearing loss ≥30 dB or tinnitus - Metallic implants such as bone plates, joint prostheses, artificial bowel outlets with magnetic closure, vascular clips, artificial heart valves, stents, or vena cava filters - Implanted electronic devices such as pacemakers or cochlear implants. |
| b. Participant information | The participant information sheet and informed consent form will be prepared in accordance with applicable regulations and submitted to the Ethics Committee of Charité for review. The informed consent discussion will be conducted by one of the study physicians listed above. If, after the oral explanation and review of the written information, the participant has no further questions and has already given verbal consent, the written informed consent form will be dated and signed by hand by both the participant and the study physician in duplicate. One copy of the signed consent form will remain at the study center, and the other copy will be given to the participant. If the participant has been given sufficient time to decide and all questions have been answered satisfactorily and completely, the screening examination may be performed on the same day. |
| 17. Recruitment | Healthy volunteers will be recruited through press releases, advertisements in daily newspapers, postings, and circular emails distributed at the ECRC, Charité, MDC, and the Berlin-Buch Health Campus. In addition, our participant database will be screened to identify suitable candidates. |
| 18. Vulnerable Populations | Not applicable. |
| 19. Relationship Between Investigator and Participants | The study physicians are not the participants’ treating physicians. |
| 20. Dependent Persons | Employees or relatives of investigators will not be enrolled. |
| 21. Participation in Other Studies | Participants will be screened for participation in other clinical studies and informed that simultaneous participation is not allowed. |
| 22. Compensation | Participants in the control group receive €200 compensation. Participants in the fasting group receive €100. |
| 23. Post‑Study Care | See section 19. |
| 24. Insurance | The study is covered by institutional liability insurance of Charité and by Accident and commuting insurance for study participants |
| 25. Documentation and Data Handling | The management of clinical data will be conducted in the form of participant files containing all source documents.  Data management for this study will be carried out in cooperation with the BIH Clinical Research Unit (CRU) Mitte. For this purpose, an electronic Case Report Form (eCRF) will be developed. Data validation procedures, including programmed automatic edit checks and consistency checks, will be implemented to ensure the validity and accuracy of the data at the time of entry into the database. Predefined eCRF entries will be reviewed by the data management team (query management). All data management procedures will be conducted in accordance with internal standard operating procedures (SOPs). After the database has been closed, the data will be converted into different data formats (e.g., CSV files) and subjected to statistical analysis. After completion or termination of the study, the data will be stored for at least 10 years. |
| 26. Health Documentation  A description of how the state of health of healthy individuals affected by the condition should be documented | All participants are assessed during the screening. Medical history, physical examination, blood tests (complete blood count, electrolytes, creatinine, transaminases, coagulation profile, lipids, fasting glucose, HbA1c), urine analysis, body weight, height, hip and waist circumference, ECG, blood pressure and heart rate are recorded in the subject file and the eCRF evaluated by the study physician. |
| 27. Adverse Events | Participants will receive comprehensive medical supervision throughout the entire study during the examination visits and will be asked about their general well-being at each visit to the study center. Before the start of the study, participants will be informed that any adverse events must be reported immediately to the responsible staff members or the study physician. This includes adverse events related to fasting or study procedures as well as symptoms that are not causally related to the study (e.g., newly occurring illnesses).  If study staff become aware of any adverse events, these must be reported immediately to the study physician, who will decide on the appropriate course of action in consultation with the principal investigator (e.g., assessment of the severity of the adverse event, treatment measures, continuation or termination of study participation).  Adverse events will be documented in a dedicated electronic Case Report Form (eCRF). |
| 28. Data Protection | The results of the study will be treated with strict confidentiality. At the beginning of the study, participants will receive an ID code that will be used to label all samples, questionnaires, documentation forms, and related materials. The ID code will not allow identification of the participant. It consists of an abbreviation of the study name (LEANER) followed by a sequential number. The assignment of names to ID codes will be known only to the principal investigator, the study physicians, and the study nurses during the course of the study. After completion of the study, the ID code list will remain with the principal investigator.  Individual data (routine laboratory values, body composition, energy expenditure, blood pressure, glucose measurements, and dietary analysis) will be provided to participants upon request. |
| 29. Confidentiality Statement | We commit to complying with all relevant data protection regulations. In particular, medical and non-medical personnel involved in the study will strictly adhere to professional confidentiality obligations.  Identifying data will be handled as described in section 28.  Data collected and biological samples obtained within the framework of this study (blood, stool, and urine samples) will be transferred to third parties (e.g., collaborating laboratories) only in encrypted or pseudonymized form. Data and biomaterials provided to third parties may be used solely for the approved research purpose and may not be forwarded to additional parties. Any material that is not used will be destroyed.  Scientific publications arising from the results of this study will be presented in a form that does not allow identification of individual participants.  Participants may withdraw their consent to further processing of their data at any time. In such a case, their stored personal data and the corresponding identification key will be deleted or destroyed. |
| 30. Study Sites and Investigators | Charité – Universitätsmedizin Berlin, Experimental and Clinical Research Center (ECRC)  Clinical Research Unit  **Principal Investigator**  • Dr. rer. medic. Anja Mähler  anja.maehler@charite.de  **Study physicians**  • Dr. Michael Boschmann  michael.boschmann@charite.de  • Dr. Nicola Wilck  nicola.wilck@charite.de  • Dr. Andras Balogh  andras.balogh@charite.de  • Dr. Hendrik Bartolomaeus  hendrik.bartolomaeus@charite.de  • Dr. Lajos Marko  [lajos.marko@charite.de](mailto:lajos.marko@charite.de)  **Scientists**  • Victoria McParland  victoria.mcparland@charite.de  • Lars Klug  lars.klug@charite.de  • PD Dr. Sylvia Bähring  sylvia.baehring@charite.de  **Study nurses**  • Gabriele Rahn  gabriele.rahn@charite.de  • Heike Schenck  heike.schenck@charite.de  **Participating laboratory**  Labor Berlin – Charité Vivantes GmbH  Sylter Straße 2  13353 Berlin  **fMRI**  NeuroCure Clinical Research Center  Dr. habil. Martin Weygandt  [martin.weygandt@charite.de](mailto:martin.weygandt@charite.de) |
| 31. Suitability of the Study Site | The Clinical Research Unit (CRU) of the Experimental and Clinical Research Center (ECRC) provides all spatial, technical, and personnel resources required for the conduct of the planned study. All staff members have received training at least once through the Charité Coordination Center for Clinical Studies (KKS) and are familiar with Good Clinical Practice (GCP) guidelines. We have been conducting GCP-compliant clinical studies for several years. The main focus of our studies includes the treatment of obesity and associated diseases (metabolic syndrome), pathophysiological and biochemical as well as cellular and molecular biological investigations in healthy volunteers, and studies in neurological diseases such as multiple sclerosis and muscle diseases such as various forms of muscular dystrophy.  Within the framework of these research activities, we have gained extensive experience in conducting clinical imaging studies, including both functional and structural MRI, in obese or eating-disordered patients^8–11^, patients with multiple sclerosis ^12–15^, patients with anxiety disorders^16^, and healthy volunteers. For the imaging components of the study, a 3T scanner (Magnetom Prisma, Siemens, Erlangen, Germany) is available at the Berlin Center for Advanced Neuroimaging (BCAN). |
| 32. Publication Policy | All data will be accessible to the principal investigator of the study.  The research results obtained will be published in scientific journals and presented at conferences. These publications will not contain any personal data that would allow identification of individual participants. |
| 33. Funding | The study is funded by research funds of the Clinical Research Unit of the ECRC. |

Fig. 1. Study design of the LEANER study with waiting-list control group

References:

1.	Wilhelmi de Toledo F, Buchinger A, Burggrabe H, Holz G, Kuhn C, Lischka E, et al. Fasting therapy - an expert panel update of the 2002 consensus guidelines. Forsch Komplementmed. 2013;20(6):434-43.

2.	Remely M, Hippe B, Geretschlaeger I, Stegmayer S, Hoefinger I, Haslberger A. Increased gut microbiota diversity and abundance of Faecalibacterium prausnitzii and Akkermansia after fasting: a pilot study. Wien Klin Wochenschr. 2015;127(9-10):394-8.

3.	Mesnage R, Grundler F, Schwiertz A, Le Maho Y, Wilhelmi de Toledo F. Changes in human gut microbiota composition are linked to the energy metabolic switch during 10 d of Buchinger fasting. J Nutr Sci. 2019;8:e36.

4.	Rubio-Aliaga I, de Roos B, Duthie SJ, Crosley LK, Mayer C, Horgan G, et al. Metabolomics of prolonged fasting in humans reveals new catabolic markers. Metabolomics. 2011;7:375-87.

5.	Lagorio CH, Madden GJ. Delay discounting of real and hypothetical rewards III: steady-state assessments, forced-choice trials, and all real rewards. Behav Processes. 2005;69(2):173-87.

6.	Doran N, Spring B, McChargue D. Effect of impulsivity on craving and behavioral reactivity to smoking cues. Psychopharmacology (Berl). 2007;194(2):279-88.

7.	Wilhelmi de Toledo F, Grundler F, Bergouignan A, Drinda S, Michalsen A. Safety, health improvement and well-being during a 4 to 21-day fasting period in an observational study including 1422 subjects. PLoS One. 2019;14(1):e0209353.

8.	Weygandt M, Schaefer A, Schienle A, Haynes JD. Diagnosing different binge-eating disorders based on reward-related brain activation patterns. Hum Brain Mapp. 2012;33(9):2135-46.

9.	Weygandt M, Mai K, Dommes E, Leupelt V, Hackmack K, Kahnt T, et al. The role of neural impulse control mechanisms for dietary success in obesity. Neuroimage. 2013;83:669-78.

10.	Weygandt M, Mai K, Dommes E, Ritter K, Leupelt V, Spranger J, et al. Impulse control in the dorsolateral prefrontal cortex counteracts post-diet weight regain in obesity. Neuroimage. 2015;109:318-27.

11.	Weygandt M, Spranger J, Leupelt V, Maurer L, Bobbert T, Mai K, et al. Interactions between neural decision-making circuits predict long-term dietary treatment success in obesity. Neuroimage. 2019;184:520-34.

12.	Weygandt M, Hackmack K, Pfuller C, Bellmann-Strobl J, Paul F, Zipp F, et al. MRI pattern recognition in multiple sclerosis normal-appearing brain areas. PLoS One. 2011;6(6):e21138.

13.	Weygandt M, Meyer-Arndt L, Behrens JR, Wakonig K, Bellmann-Strobl J, Ritter K, et al. Stress-induced brain activity, brain atrophy, and clinical disability in multiple sclerosis. Proc Natl Acad Sci U S A. 2016;113(47):13444-9.

14.	Weygandt M, Wakonig K, Behrens J, Meyer-Arndt L, Soder E, Brandt AU, et al. Brain activity, regional gray matter loss, and decision-making in multiple sclerosis. Mult Scler. 2018;24(9):1163-73.

15.	Weygandt M, Behrens J, Brasanac J, Soder E, Meyer-Arndt L, Wakonig K, et al. Neural mechanisms of perceptual decision-making and their link to neuropsychiatric symptoms in multiple sclerosis. Mult Scler Relat Disord. 2019;33:139-45.

16.	Weygandt M, Blecker CR, Schafer A, Hackmack K, Haynes JD, Vaitl D, et al. fMRI pattern recognition in obsessive-compulsive disorder. Neuroimage. 2012;60(2):1186-93.

Name and signature of the applicant(s):

I hereby certify that the information provided in this application is correct. I am of the opinion that it is possible to carry out the above-mentioned study in accordance with the protocol and national legislation.

Name: Dr Mähler

First name: Anja

Work address: Lindenberger Weg 80, 13125 Berlin

Position: Research Assistant / Study Coordinator

Date: 6 May 2020
